# Coherent dynamics of multi-spin V${}_{{{{{{{{\rm{B}}}}}}}}}^{-}$ center in hexagonal boron nitride

**DOI:** 10.1038/s41467-022-33399-2

**Published:** 2022-09-29

**Authors:** Wei Liu, Viktor Ivády, Zhi-Peng Li, Yuan-Ze Yang, Shang Yu, Yu Meng, Zhao-An Wang, Nai-Jie Guo, Fei-Fei Yan, Qiang Li, Jun-Feng Wang, Jin-Shi Xu, Xiao Liu, Zong-Quan Zhou, Yang Dong, Xiang-Dong Chen, Fang-Wen Sun, Yi-Tao Wang, Jian-Shun Tang, Adam Gali, Chuan-Feng Li, Guang-Can Guo

**Affiliations:** 1 CAS Key Laboratory of Quantum Information, University of Science and Technology of China, Hefei, P. R. China; 2 CAS Center For Excellence in Quantum Information and Quantum Physics, University of Science and Technology of China, Hefei, 230026 P. R. China; 3 Hefei National Laboratory, University of Science and Technology of China, Hefei, 230088 China; 4 Max-Planck-Institut für Physik komplexer Systeme, Nöthnitzer Street 38, D-01187 Dresden, Germany; 5 Department of Physics, Chemistry and Biology, Linköping University, SE-581 83 Linköping, Sweden; 6 Wigner Research Centre for Physics, PO Box 49, H-1525 Budapest, Hungary; 7 Department of Atomic Physics, Institute of Physics, Budapest University of Technology and Economics, Műegyetem rakpart 3., H-1111 Budapest, Hungary

**Keywords:** Quantum metrology, Two-dimensional materials, Optical properties of diamond

## Abstract

Hexagonal boron nitride (hBN) has recently been demonstrated to contain optically polarized and detected electron spins that can be utilized for implementing qubits and quantum sensors in nanolayered-devices. Understanding the coherent dynamics of microwave driven spins in hBN is of crucial importance for advancing these emerging new technologies. Here, we demonstrate and study the Rabi oscillation and related phenomena of a negatively charged boron vacancy (V${}_{{{{{{{{\rm{B}}}}}}}}}^{-}$) spin ensemble in hBN. We report on different dynamics of the V${}_{{{{{{{{\rm{B}}}}}}}}}^{-}$ spins at weak and strong magnetic fields. In the former case the defect behaves like a single electron spin system, while in the latter case it behaves like a multi-spin system exhibiting multiple-frequency dynamical oscillation as beat in the Ramsey fringes. We also carry out theoretical simulations for the spin dynamics of V${}_{{{{{{{{\rm{B}}}}}}}}}^{-}$ and reveal that the nuclear spins can be driven via the strong electron nuclear coupling existing in V${}_{{{{{{{{\rm{B}}}}}}}}}^{-}$ center, which can be modulated by the magnetic field and microwave field.

## Abstract (web summary)

Understanding the coherent dynamics of electron and nucleus spins in hBN is crucial for their applications as qubits and quantum sensors. Here the authors report room-temperature coherent manipulation of the negatively charged boron vacancy spins in hBN and study their dynamics under weak and strong magnetic fields.

## Introduction

Van der Waals (vdW) materials exhibit diverse electronic properties from semimetal (e.g., graphene) through semiconductor (e.g., transition metal dichalcogenides, TMDCs for short) to insulator (e.g., hexagonal boron nitride, or hBN)^1^. Their common feature is the layered structure, namely, the intralayer atoms are combined by strong chemical bonds while the layers are connected by the relatively weak vdW force. This feature makes the layers of different vdW materials easy to be stacked together to form heterostructures^2^, which have the advantage of no lattice mismatch compared to their three-dimensional counterparts, including GaAs, silicon, or diamond, etc. Besides, vdW materials have strong interaction with light due to the two-dimensional confinement of the electronic states^3^. These characteristics lead to a great variety of applications of vdW materials, such as photocurrent generation^4^, light-emitting diode^5^, field effect transistor^6^, single photon^7–26^ and optical parametric amplification^3^. Moreover, the light-valley interaction in TMDCs leads to the field of valleytronics^27,28^. All these applications will expectedly contribute to the design and construction of photonic and electronic devices in nanoscale benefiting from the atomic thickness of vdW materials.

Among this family of layered materials, hBN has a large bandgap of ~6 eV, which makes it possible to host plenty of defect states in the bandgap similar to diamond^29–31^ and silicon carbide^32–34^. Single-layer hBN was first found to host room-temperature quantum emitter in 2016 by ref. 15, that stimulated numerous works to explore promising quantum emitters^16–25^ and potential solid spin qubits^35–43^. Quantum emitters in hBN (in monolayer, flake or bulk) have the advantages of high brightness^21,24^, broad spectral range^20^, easy tunability^21,22^, and easy fabrication. A diverse set of fabrication methods has been demonstrated, such as chemical etching^18^, electron or ion irradiation^18,19,25,40^, laser ablation^19,41^ and strain^23^.

Recently, defects in hBN have attracted a lot of attentions as a good candidate for solid spin qubit (particularly, in the vdW-nano-devices). Electron paramagnetic resonance (EPR) signals in hBN have been found in very early decades^44–46^, whose origins have been identified by numerical calculations recently^47,48^. The theoretical works have also predicted many possible defects in hBN that can give rise to optically detected magnetic resonance (ODMR) signals. In experiment, Exarhos et al.^35^ reported on magnetic-field-dependent fluorescence intensity of a hBN defect in 2019. Later, related ODMR signals were revealed by Gottscholl et al.^36,37^, Chejanovsky et al.^38^, and Mendelson et al.^39^, the defects were tentatively assigned to be V${}_{{{{{{{{\rm{B}}}}}}}}}^{-}$ (negatively charged boron vacancy) defects^36,37^ or the defects related to carbon^39^. After these initial experimental results, several theoretical analyzations were carried out, especially for V${}_{{{{{{{{\rm{B}}}}}}}}}^{-}$ defects^49,50^. Furthermore, the temperature-dependent features of V${}_{{{{{{{{\rm{B}}}}}}}}}^{-}$ spin defect have also been investigated experimentally^42^. Here, we demonstrate room-temperature coherent control of a V${}_{{{{{{{{\rm{B}}}}}}}}}^{-}$ spin ensemble in hBN. Especially, the Rabi oscillations under different magnetic fields indicate that a strong electron nuclear spin coupling exists in V${}_{{{{{{{{\rm{B}}}}}}}}}^{-}$ center. We also carry out the theoretical simulations of ${\mathrm V}_{{{{{{{{\rm{B}}}}}}}}}^{-}$ spin dynamics and reveal that the nuclear spins can be modulated by both the microwave (MW) and the magnetic fields further giving rise to multi-spin dynamics of the ${\mathrm V}_{{{{{{{{\rm{B}}}}}}}}}^{-}$ center.

## Results

### Sample description

The hBN sample we investigated here is a bulk synthetic monocrystalline flake with the ~1 mm lateral size purchased from HQ Graphene. The sample is irradiated by neutron flow with a thermal flux of 1.4 × 10^13^ cm^−2^s^−1^ for 4 h in a nuclear reactor (integrated dose ~2 × 10^17^ cm^−2^), similar to that in ref. 36, to generate the V${}_{{{{{{{{\rm{B}}}}}}}}}^{-}$ defects. The photoluminescence (PL) of the irradiated hBN flake is characterized by a broadband spectrum centering around ~ 800 nm (See Supplementary Note 2), that is consistent with the previously reported theoretical and experimental PL spectra of the ${\mathrm V}_{{{{{{{{\rm{B}}}}}}}}}^{-}$ defect^37,49,51^.

**Fig. 1:**
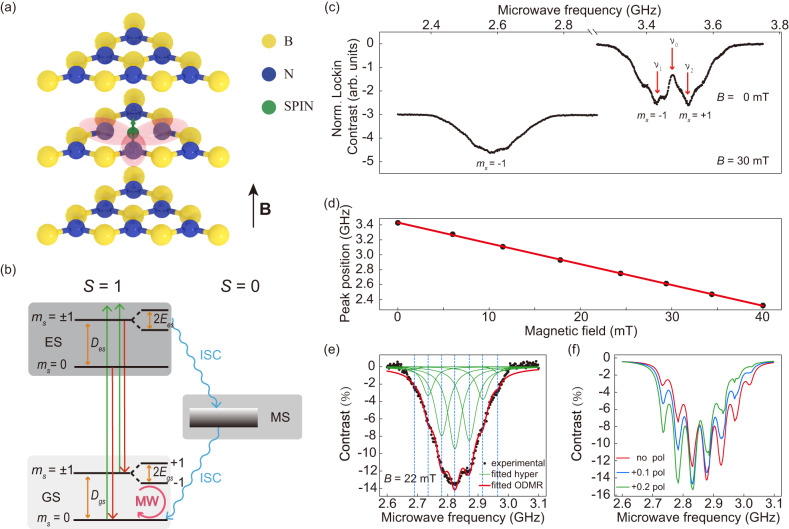
Simplified atomic structure, energy levels and ODMR results of V${}_{{{{{{{{\rm{B}}}}}}}}}^{-}$ center. **a** Geometrical structure of V${}_{{{{{{{{\rm{B}}}}}}}}}^{-}$ defect in multilayer hBN flake with alternating boron (yellow), nitrogen (blue) atoms and a negatively charged boron vacancy (green arrows). The negatively charged boron vacancy comprises a missing boron atom and an extra electron surrounded by three equivalent nitrogen atoms. The magnetic field B applied in the experiment is perpendicular to the two-dimensional plane of hBN, that is, parallel to the hexagonal *c* axis. **b** Simplified energy levels of V${}_{{{{{{{{\rm{B}}}}}}}}}^{-}$ center and the related radiative transitions (red) and non-radiative inter-system crossing (ISC) transition (blue) among ground states (GS), excited states (ES) and metastable states (MS). The 532-nm laser (green) is used for the spin polarization and readout and the microwave (pink) is used for coherent control of the spin state. **c** Room-temperature ODMR spectra measured at 0-mT (top pane) and 30-mT (lower) magnetic fields. At 0 mT, the ODMR spectrum is fitted by a two Lorentzian function to obtain the energy-level splittings *ν*_1_ ~ 3.424 GHz and *ν*_2_ ~ 3.533 GHz. **d** Dependence of the *m*_*s*_ = − 1 ↔ *m*_*s*_ = 0 splitting shift on magnetic field, from which we obtain the *g* factor of V${}_{{{{{{{{\rm{B}}}}}}}}}^{-}$ spin to be 1.992 ± 0.010. **e** Hyperfine structure of the ODMR spectrum measured at *B* = 22 mT, fitted with a seven Lorentzian function. The solid red curve is fitting envelope and solid green curves are fitting Lorentzian components. The dashed lines marked the seven hyperfine peaks with a characteristic splitting of *A* ~ 45.8 MHz. **f** Theoretical ODMR spectra as *B* = 21.7 mT for nonpolarized (red) and polarized (blue and green) nearest neighbor ^14^N nuclear spins, where label “*x* pol” means the nuclear populations are 1/3 + *x*, 1/3, and 1/3-*x* on the *m*_*I*_ = +1, 0, and −1 nuclear-spin states, respectively. The nuclear population of *m*_*I*_ = 0 is fixed as 1/3 in consideration that the *m*_*I*_ = 0 population always changes little, compared with the *m*_*I*_ = ± 1 populations, under modulations of magnetic and microwave fields.

The atomic structure of V${}_{{{{{{{{\rm{B}}}}}}}}}^{-}$ defect is shown in Fig. 1a and a simplified diagram of the energy levels of the V${}_{{{{{{{{\rm{B}}}}}}}}}^{-}$ is sketched in Fig. 1b. As discussed in refs. 37,49,51,52, the V${}_{{{{{{{{\rm{B}}}}}}}}}^{-}$ defect exhibits a triplet ground state (GS), which is primarily constituted of three energy levels with *m*_*s*_ = 0 and *m*_*s*_ = ±1, and *D* is the zero-field splitting (ZFS) between them. ES and MS represent the triplet (*S* = 1) excited state and the metastable singlet (*S* = 0) state, respectively. The green arrows represent the laser excitation which pump the population to ES, while the red arrows represent the fluorescence to be detected. The blue wavy arrows represent inter-system crossings (ISC) between *S* = 1 and *S* = 0 states. The pink circled arrow is the applied microwave (MW) between *m*_*s*_ = 0 and *m*_*s*_ = ±1, which will modulate the populations of these states and hence change the intensity of the fluorescence. By recording the difference of the fluorescence intensities, we can read-out the state of the spin qubit. This method is called ODMR. We perform ODMR measurements to further confirm the origin and character of the generated spin defect in our hBN sample.

### ODMR results

By sweeping the frequency of the MW field, typical ODMR spectra of the V${}_{{{{{{{{\rm{B}}}}}}}}}^{-}$ in hBN at zero and 30-mT magnetic fields are obtained, see Fig. 1c. Here the excitation laser is always on and the MW operates at on/off mode. The contrast is obtained from the difference between the MW-on and MW-off fluorescences. The frequency *ν*_0_ = (*ν*_1_ + *ν*_2_)/2 = 3.479 GHz corresponds to the ZFS *D*, where *ν*_1_ (*ν*_2_) corresponds to the energy-level splittings between *m*_*s*_ = −1 (*m*_*s*_ = +1) and *m*_*s*_ = 0 states. We also observe the frequency shift with the magnetic field for the *m*_*s*_ = −1 ↔ *m*_*s*_ = 0 transition and fit the *g*-factor of this spin to be 1.992 ± 0.010 as shown in Fig. 1d (See more ODMR spectra in Supplementary Note 3). Remarkably, for each transition peak of *m*_*s*_ = ±1 to *m*_*s*_ = 0, we can clearly see several hyperfine peaks, which should be related to the electron nuclear hyperfine interaction.

### Hyperfine structures

For the V${}_{{{{{{{{\rm{B}}}}}}}}}^{-}$ center in hBN, there are three nearest neighbor nitrogen nuclei (^14^N with 99.6% abundance) surrounding the boron vacancy site, each of which has a nuclear spin of *I* = 1. Then the electron spin and the three ^14^N nuclear spins are coupled and form a 4-spin system whose atomic structure is marked by the red lobes in Fig. 1a, and a total of 2(3*I*) + 1 = 7 hyperfine transitions should be observed in the ODMR spectra. To confirm this, we carry out theoretical simulation for the 4-spin V${}_{{{{{{{{\rm{B}}}}}}}}}^{-}$ system and obtain the theoretical ODMR spectra at 21.7-mT magnetic field. The theoretical ODMR spectra exhibit seven distinct hyperfine peaks with the hyperfine splitting of ~48 MHz. The polarization of ^14^N nuclear spin can generate an asymmetric ODMR spectra as shown in Fig. 1f. Further, we fit the experimental ODMR spectra with seven Lorentzian functions as shown in Fig. 1e. The experimental hyperfine splitting is ~45 MHz, which is consistent with our theoretical results and the previously reported works of V${}_{{{{{{{{\rm{B}}}}}}}}}^{-}$ spin^37,49^. Considering the fact that the ODMR hyperfine structure depends on the intrinsic electron nuclear structure, the generated defects in this work can be identified as the V${}_{{{{{{{{\rm{B}}}}}}}}}^{-}$ defect. In addition, the observed ODMR spectra in Fig. 1c, e also exhibit asymmetric hyperfine structures indicating the polarization of ^14^N nuclear spins occurring in V${}_{{{{{{{{\rm{B}}}}}}}}}^{-}$ center under the MW drive. By studying the hyperfine structures of more ODMR spectra at different magnetic fields, we find that the ^14^N-spin polarization is enhanced with the increase of external magnetic field (See Supplementary Note 3).

### Rabi oscillations

**Fig. 2:**
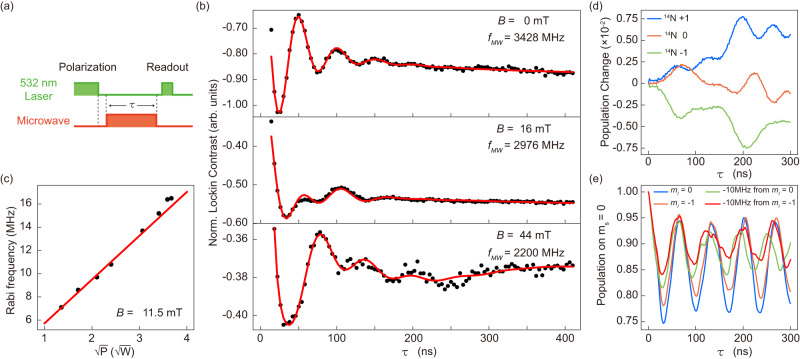
Rabi oscillations. **a** Pulse sequence of Rabi measurement comprising of a first laser pulse for spin polarization, then a microwave pulse with length *τ* for spin manipulation, and finally a second laser pulse for state readout. **b** Room-temperature Rabi oscillations on the *m*_*s*_ = −1 ↔ *m*_*s*_ = 0 transition observed at different magnetic fields of *B* = 0 mT (top), *B* = 16 mT (middle) and *B* = 44 mT (bottom). The data are fitted using $a{e}^{-\tau /{T}_{a}}\mathop{\prod }\nolimits_{i=1}^{n}\cos (2\pi {f}_{i}\tau+{\phi }_{i})+b{e}^{-\tau /{T}_{b}}+c$ with the amount of the different oscillation components are chosen empirically as *n* = 1 for *B* = 0 mT, *n* = 2 for *B* = 16 mT, and *n* = 3 for *B* = 44 mT (red curves). The fitting parameters *a*, *T*_*a*_, *f*_*i*_, *ϕ*_*i*_, *b*, *T*_*b*_ and *c* are oscillation amplitude, oscillation decay time, frequency, phase, background decay amplitude, background decay time, and constant background, respectively. **c** Linear dependence of Rabi frequency on the square root of microwave power $\sqrt{P}$. **d** Theoretical dynamical oscillation of ^14^N nuclear spins with the driving MW resonant at the *m*_*I*_ = − 1 hyperfine peak. The blue, orange, and green curves show the dynamical polarization changes on the *m*_*I*_ = +1, 0, and −1 nuclear spin states under continuous MW driving, respectively. **e** Theoretical Rabi oscillation of the 4-spin V${}_{{{{{{{{\rm{B}}}}}}}}}^{-}$ system. The blue, orange, green, and red curves show Rabi oscillations driven by the MW with frequencies at center hyperfine peak, *m*_*I*_ = − 1 hyperfine peak, −10 MHz detuning from *m*_*I*_ = 0 hyperfine peak, and -10 MHz detuning from *m*_*I*_ = − 1 hyperfine peak, respectively.

The next step is to coherently manipulate the defect spin for which the Rabi oscillation is a key tool. Here, we utilize the two-level electronic states *m*_*s*_ = { − 1, 0} as the electron spin qubit to perform the coherent control. The time diagram of the MW and the laser pulse sequences are shown in Fig. 2a. After a long excitation-laser pulse, the spin is polarized to *m*_*s*_ = 0 state, then a MW pulse with a length of *τ* is applied to rotate the spin, followed by a readout laser pulse. The detected Rabi oscillations are shown in Fig. 2b. At magnetic field of *B* = 0 mT, we see a standard decaying Rabi oscillation, but we also observe a tiny decay of background. By varying the MW power, we derive the linear dependence of the fitted Rabi frequency versus the square root of MW power $\sqrt{P}$ (see Fig. 2c), which indicates the validity of our Rabi results. Remarkably, for the results of non-zero magnetic field, we observe the Rabi oscillations containing multiple-frequency components and the fitted relaxation time of oscillation seems increasing with the magnetic field. Here, we fit the observed Rabi oscillations by a product summation of multiple oscillation functions^53^, with the amount of oscillation components are chosen empirically as *n* = 1, 2 and 3 for different magnetic fields (See Supplementary Note 4). To reveal the mechanism of the multiple-frequency Rabi oscillation, we carry out the theoretical simulation for the spin dynamics of the closed 4-spin V${}_{{{{{{{{\rm{B}}}}}}}}}^{-}$ system. Our simulation results indicate that the detuning of the MW frequency can drive dynamically the ^14^N nuclear spins and lead to the ^14^N polarization oscillation as shown in Fig. 2d. Then the MW-driven ^14^N nuclear spin dynamics can modulate the Rabi-oscillation curve, as the simulation results shown in Fig. 2e, where even a slight detuning of 10 MHz of the MW can induce multiple-frequency oscillation curve. At strong magnetic field, the ^14^N nuclear spins tend to be polarized, as we demonstrated by our ODMR results, and the relaxation effect seems weaken according to the fitting results of Rabi oscillation in Fig. 2b (See Supplementary Note 4). Hence the ^14^N nuclear spins tend to be driven by detuning MW at strong magnetic field and lead to the multiple-frequency Rabi oscillations of V${}_{{{{{{{{\rm{B}}}}}}}}}^{-}$ spin. In addition, according to the theoretical simulation results taking many-body spin bath into account, we conclude that the nuclear spin bath surrounding V${}_{{{{{{{{\rm{B}}}}}}}}}^{-}$ is responsible for the decay of Rabi oscillation, while the nearest neighbor ^14^N nuclear spins are responsible for the modulation of multiple-frequency Rabi oscillation and the decay of background (See Supplementary Note 7).

### *T*_1_ measurement and spin echo detections

**Fig. 3:**
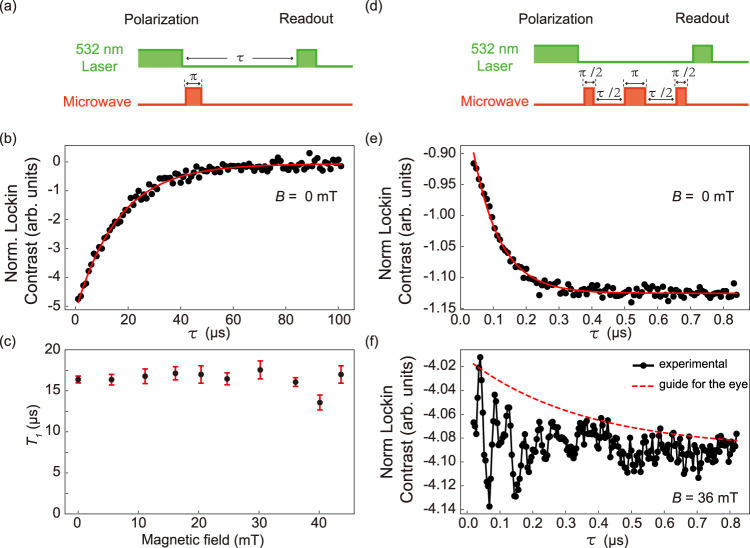
*T*_1_ measurement and spin echo detections. **a** Pulse sequence for characterizing the spin-lattice relaxation dynamics, including the spin polarization and read-out laser pulse, the *π*-pulse microwave obtained from Rabi measurement and the varying free evolution time *τ*. **b** *T*_1_ measurement at 0 mT revealing the spin-lattice relaxation time of *T*_1_ = 16.377 ± 0.416 μs. **c** *T*_1_ time versus magnetic field, suggesting that there is roughly no *T*_1_ dependence on magnetic field. The error bars correspond to the fitting errors. **d** Pulse sequence for spin echo measurement with $\frac{\pi }{2}-\frac{\tau }{2}-\pi -\frac{\tau }{2}-\frac{\pi }{2}$ sequence, where *τ* is the free evolution time. **e** Optically-detected spin-echo measurement at 0 mT. **f** Spin echo at 36 mT, which cannot be fitted well, showing complicated oscillations induced by the nuclear spin bath and the red line is only a guide for the eye.

With the obtained Rabi frequency, we can define $\frac{\pi }{2}$-pulse and *π*-pulse. Utilizing a *π*-pulse, we can measure the spin-lattice relaxation time *T*_1_. The pulse sequence is shown in Fig. 3a, while the relaxation result at *B* = 0 mT is depicted in Fig. 3b. By fitting this result, we obtain *T*_1_ = 16.377 ± 0.416 μs. Then we repeat this sequence for various magnetic fields and obtain the results in Fig. 3c. We find *T*_1_ is approximately independent of the magnetic field. Next, we perform the sequence $\frac{\pi }{2}-\frac{\tau }{2}-\pi -\frac{\tau }{2}-\frac{\pi }{2}$ (spin echo, see Fig. 3d) to measure *T*_2_. At *B* = 0 mT, we observe a monotonic decay of contrast shown in Fig. 3e and *T*_2_ is fitted to be 82.121 ± 2.462 ns, which is quite short but in good agreement with ref. 54. At *B* = 36 mT, we find the decayed-contrast curve is complicatedly modulated (see Fig. 3f). We cannot fit it well and the red line is only a guide for the eye. Here we note that, since *T*_2_ is quite short, the impact of the MW-pulse lengths, especially the *π*-pulse length (~26 to 44 ns in this work), cannot be ignored, therefore, the values of the fitted *T*_2_ may be affected, however the order of magnitude can be determined. For *T*_1_, which is much longer than the MW-pulse lengths, this is not an issue.

### Ramsey interference

**Fig. 4:**
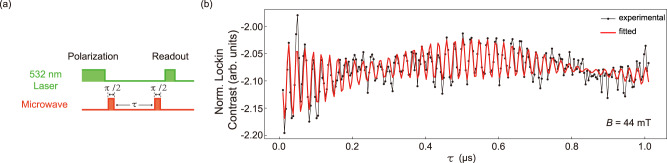
Ramsey interference. **a** Ramsey pulse sequence with $\frac{\pi }{2}-\tau -\frac{\pi }{2}$. **b** Ramsey result at 44-mT magnetic field driven by 2200-MHz microwave starting from *τ* = 10 ns. The red curve is the fitting result using the empirical fitting function $\mathop{\sum }\nolimits_{i=-1}^{1}{a}_{i}{e}^{-\tau /{T}_{2i}^{*}}\cos (2\pi {f}_{i}\tau+{\phi }_{i})+b$. The fitting parameters *a*_*i*_, ${T}_{2i}^{*}$, *f*_*i*_, *ϕ*_*i*_ and *b* are amplitude, oscillation decay time, frequency, phase, and constant background, respectively. Three frequencies *f*_−1_, *f*_0_ and *f*_+1_ are observed and two of them form a clear beat. The distances between the adjacent frequencies are both around ~45 MHz. The fitted ${T}_{2}^{*}$ corresponding to these three frequencies are 0.665 ± 0.108 μs, 2.500 ± 2.160 μs and 1.448 ± 0.841 μs, respectively.

We also perform Ramsey interference experiment on V${}_{{{{{{{{\rm{B}}}}}}}}}^{-}$ spins. The pulse sequence is presented in Fig. 4a. We observe no oscillations but a monotonic decay at weak magnetic fields (See Supplementary Note 6), which may be caused by the fast relaxation due to the nuclear spin bath, corresponding to a short ${T}_{2}^{*}$. In contrast, when we apply a magnetic field of *B* = 44 mT and set the MW frequency at *f*_MW_ = 2200 MHz, we see a multiple-frequency oscillation, in which a beat is clearly recognized, and it is superposed on another slow oscillation. These three frequencies are fitted as *f*_−1_ = −44.171 ± 0.039 MHz, *f*_0_ = 0.934 ± 0.131 MHz, *f*_1_ = 45.872 ± 0.063 MHz, respectively, and the distance between the adjacent frequencies are *f*_0_ − *f*_−1_ = 45.105 ± 0.136 MHz ≈ *f*_1_ − *f*_0_ = 44.938 ± 0.145 MHz.

Similar to the results of Rabi oscillations, the nearest neighbor ^14^N nuclear spins are driven by the MW at strong magnetic field and the observed multiple frequencies in Ramsey interference correspond to spin-rotation frequencies in rotating frame on three hyperfine levels. The frequency *f*_0_ is the detuning between MW and center hyperfine level, as well as *f*_−1_ and *f*_1_ are the MW detuning from two adjacent nonzero hyperfine levels. In addition, the fitted ${T}_{2}^{*}$ of these three hyperfine levels are 0.665 ± 0.108 μs, 2.500 ± 2.160 μs and 1.448 ± 0.841 μs, respectively. It seems that the magnetic field suppresses the spin relaxation of V${}_{{{{{{{{\rm{B}}}}}}}}}^{-}$ center.

### Spin-bath relaxation

**Fig. 5:**
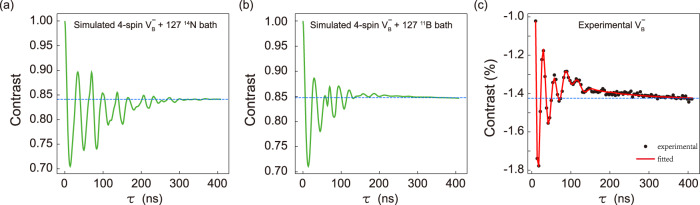
Spin dynamics of open V${}_{{{{{{{{\rm{B}}}}}}}}}^{-}$ system with relaxation from the many-body nuclear spin bath at *B* = 21.7 mT. **a**, **b** Theoretical Rabi oscillation interacted with the surrounding 127 ^14^N or ^11^B as the many-body spin bath. The dashed lines are provided as a baseline of long-time background. **c** Experimental Rabi oscillation driven by 2820 MHz microwave at *B* = 21.7 mT. The red curve is the fitting result using the multiple-frequency fitting function similar to that used in Fig. 2b, where the amount of different oscillation components is chosen empirically as *n* = 3.

To study the spin relaxation mechanism of V${}_{{{{{{{{\rm{B}}}}}}}}}^{-}$ center, we carry out theoretical simulation for an open V${}_{{{{{{{{\rm{B}}}}}}}}}^{-}$ system, in which many ^14^N or ^11^B nuclear spins are taken into account to describe the spin bath surrounding the 4-spin V${}_{{{{{{{{\rm{B}}}}}}}}}^{-}$ center in hBN. Cluster approximation and an extended Lindbladian method are used to simulate the V${}_{{{{{{{{\rm{B}}}}}}}}}^{-}$ spin dynamics^55^. Here, we consider a spin bath containing 127 ^14^N or 127 ^11^B nuclear spins and acquire the Rabi oscillation of V${}_{{{{{{{{\rm{B}}}}}}}}}^{-}$ spin with the spin-bath relaxation at a *B* = 21.7 mT. The simulation results of the ^14^N and ^11^B spin bathes are shown in Fig. 5a, b, respectively. The two simulation results are similar, however, there are also significant differences. In the ^14^N spin bath, the Rabi oscillation decays on a longer timescale, while the oscillation’s baseline exhibits the obvious positive offset. To verify which spin bath contributes mainly to the relaxation of V${}_{{{{{{{{\rm{B}}}}}}}}}^{-}$ spin, we compare the two theoretical results with the experimental Rabi oscillation at the same magnetic field (Fig. 5c). One can see that the major characteristics, like the decay timescale and oscillation’s baseline, are captured by the ^11^B spin bath. Considering the simulation results of the closed V${}_{{{{{{{{\rm{B}}}}}}}}}^{-}$ center without including a spin bath we observe no decay for long timescale (Fig. 2e). Therefore, we conclude that the ^11^B-nuclear spin bath is the main source for the relaxation of V${}_{{{{{{{{\rm{B}}}}}}}}}^{-}$ spin dynamics.

We found related works were finished by Gottscholl et al.^43^, Haykal et al.^54^ and Gao et al.^56^. Our results partially coincide with theirs, e.g., the room-temperature *T*_1_ and *T*_2_ at zero magnetic field, which validate the results of each other. On the other hand, we note most results in this work are different from theirs, e.g., the magnetic-field-dependent behaviors, the multiple-frequency oscillations in Rabi and Ramsey results, especially the beat. These different results in these papers complement to each other, and exhibit a more comprehensive knowledge of V${}_{{{{{{{{\rm{B}}}}}}}}}^{-}$ spin defect in hBN.

## Discussions

The domination of ^11^B nuclear spin for the relaxation effect can be due to the strong hyperfine couplings of ^11^B nuclear spins beyond the nearest neighbor ^14^N nuclear spins, as well as the possible low polarization of ^11^B spins. The inter-nuclear ^11^B-^11^B coupling is ~20 times stronger than the ^14^N-^14^N coupling, resulting in a fast spin diffusion in the boron sublattice. Besides, we show that the polarization of the nuclear-spin bath in V${}_{{{{{{{{\rm{B}}}}}}}}}^{-}$ can be enhanced by the external magnetic field, then the spin-bath relaxation process like nuclear flip-flop process is suppressed^57–60^. Hence at strong magnetic field, the Rabi oscillation and Ramsey interference exhibit enhanced coherent features of the multiple-frequency components and are modulated obviously by the MW-driven nuclear spins via the strong electron nuclear coupling in V${}_{{{{{{{{\rm{B}}}}}}}}}^{-}$ center. In addition, the asymmetry of the ODMR spectra corresponding to the nuclear-spin polarization is also enhanced at the high magnetic fields. It indicates that the nearest neighbor ^14^N nuclear spin in V${}_{{{{{{{{\rm{B}}}}}}}}}^{-}$ center could be explored as another controllable spin in hBN.

As the main candidate for spin qubit in vdW material (to date), the coherent operations of defects in hBN based on Rabi oscillation play a crucial role and provide a powerful tool for the design and construction of spin-based vdW-nano-devices, especially when different techniques of vdW heterostructures are combined. Although *T*_2_ seems to be still quite short, there will be several methods to improve it. For example, the short *T*_2_ should be related to the relaxation of nuclear spin bath driven mainly by the Fermi contact interaction, and also reduced by the dark electron spin impurities^54^. The higher external magnetic field or decreased neutron-irradiation would be helpful to reduce the coherence relaxation. A suitable annealing on the hBN sample could be also explored since the high-temperature condition can reduce the V${}_{{{{{{{{\rm{B}}}}}}}}}^{-}$ defect number. In addition, putting the sample into a low-temperature cryostat can also reduce the lattice relaxation.

In summary, we demonstrated the room-temperature coherent manipulation and Rabi oscillation of V${}_{{{{{{{{\rm{B}}}}}}}}}^{-}$ spins in hBN, based on which we also detected *T*_1_ and performed the spin-echo and Ramsey-interference experiments. We find *T*_1_ is almost not affected by the magnetic field, however, the results of the Rabi oscillation, spin echo, and Ramsey oscillation are very different under the conditions of weak and relatively strong magnetic fields. To reveal the intrinsic mechanism behind the experimental measurements, we also carried out theoretical simulations on 4-spin V${}_{{{{{{{{\rm{B}}}}}}}}}^{-}$ systems and obtain the theoretical ODMR spectra, nuclear-spin dynamics, and spin-bath relaxation. The theoretical and experimental results are basically consistent with each other and reveal the strong electron nuclear coupling existing in V${}_{{{{{{{{\rm{B}}}}}}}}}^{-}$ center. The ^14^N nuclear spins can be driven by the MW and polarized at strong magnetic field. The ^11^B nuclear spin bath in hBN should dominate the relaxation of V${}_{{{{{{{{\rm{B}}}}}}}}}^{-}$ spin dynamics. The strong magnetic field can reduce the spin relaxation and enhance the MW-driven nuclear-spin oscillation to modulate V${}_{{{{{{{{\rm{B}}}}}}}}}^{-}$ spin dynamics.

## Methods

### Experimental setup

A home-built confocal microscopy system combined with a MW system is used for the spin manipulation measurements. A 532-nm laser modulated by an acousto-optic modulator (AOM) is focused on the sample through a 100 × objective (N.A. = 0.9) for the spin initialization and excitation. The photoluminescence with wavelength above 750 nm is collected by the same objective and guided through a multimode fiber to a photoreceiver with high gain (~10^10^) or an avalanche photodiode (APD) for signal readout. The MW generated by a synthesized signal generator is radiated through a 20-μm diameter copper wire close to the sample or a gold film microwave stripline plated on the substrate and controlled by a switch. To manipulate the laser and MW, a pulse blaster card is exploited to produce the corresponding electrical pulse sequences. A lock-in amplifier or a data acquisition card is finally used to analyze and extract the signal of spin state. For the magnetic field, an electromagnet is placed directly below the sample to generate a *c*-axis field. See Supplementary Note 1 for more details of the experimental setup.

### Simulation methodology

In the theoretical calculations, we study the dynamics of microwave-driven V${}_{{{{{{{{\rm{B}}}}}}}}}^{-}$ spin systems by two simulation methods. (1) For the closed 4-spin V${}_{{{{{{{{\rm{B}}}}}}}}}^{-}$ model, we apply the exact time evolution of a 4-spin model, consisting of the center 1-spin electronic spin and its three nearest neighbor 1-spin ^14^N nuclear spins. All the hyperfine parameters are taken from our ab initio electronic structure calculations. See Supplementary Note 7 for the details of Hamiltonian parameters of V${}_{{{{{{{{\rm{B}}}}}}}}}^{-}$ model. (2) For the open 4-spin V${}_{{{{{{{{\rm{B}}}}}}}}}^{-}$ model, we consider an additional many-body spin bath containing 127 ^14^N or ^11^B nuclear spins, which are the second and farther neighbor shells of the V${}_{{{{{{{{\rm{B}}}}}}}}}^{-}$ center in hBN. The many-body spin bath interacts with the 4-spin V${}_{{{{{{{{\rm{B}}}}}}}}}^{-}$ system which is described by a cluster approximation combined with an extended Lindbladian method developed in ref. 55. In the cluster approximation, the total system is divided into 127 subsystem, and every subsystem contains the 4-spin V${}_{{{{{{{{\rm{B}}}}}}}}}^{-}$ system and one of the nuclear spins in the many-body spin bath. In contrast to method (1), method (2) can induce the relaxation effects in a parameter-free manner. No additional decoherence and relaxation effects are included beyond the cluster approximation in the simulations. In both methods, the microwave field is added with no approximation, i.e., an oscillating magnetic field with in-plane magnetic field polarization is added to the model to describe the external drive. This allows us to account for the dressing and multi-spin resonances^55^. It should be noted that the theoretical calculations applied in this work are parameter free, i.e., no experimental or adjustable parameters are used in the calculations. Hence the theoretical and experimental results are independent of each other.

## Supplementary information

Supplementary Information

## Data Availability

The data that support the findings of this study are available within the article and its Supplementary Information. Additional relevant data are available from the corresponding authors upon reasonable request.

## Code availability

The code used in this study is available from the corresponding authors upon reasonable request.
