# Author Correction: Coherent dynamics of multi-spin V${}_{{{{{{{{{{\rm{B}}}}}}}}}}}^{-}$ center in hexagonal boron nitride

**DOI:** 10.1038/s41467-023-39331-6

**Published:** 2023-06-14

**Authors:** Wei Liu, Viktor Ivády, Zhi-Peng Li, Yuan-Ze Yang, Shang Yu, Yu Meng, Zhao-An Wang, Nai-Jie Guo, Fei-Fei Yan, Qiang Li, Jun-Feng Wang, Jin-Shi Xu, Xiao Liu, Zong-Quan Zhou, Yang Dong, Xiang-Dong Chen, Fang-Wen Sun, Yi-Tao Wang, Jian-Shun Tang, Adam Gali, Chuan-Feng Li, Guang-Can Guo

**Affiliations:** 1 CAS Key Laboratory of Quantum Information, University of Science and Technology of China, Hefei, PR China; 2 CAS Center for Excellence in Quantum Information and Quantum Physics, University of Science and Technology of China, Hefei, 230026 PR China; 3 Hefei National Laboratory, University of Science and Technology of China, Hefei, 230088 China; 4 Max-Planck-Institut für Physik komplexer Systeme, Nöthnitzer Street 38, D-01187 Dresden, Germany; 5 Department of Physics, Chemistry and Biology, Linköping University, SE-581 83 Linköping, Sweden; 6 Wigner Research Centre for Physics, PO Box 49, H-1525 Budapest, Hungary; 7 Department of Atomic Physics, Institute of Physics, Budapest University of Technology and Economics, Műegyetem rakpart 3., H-1111 Budapest, Hungary

**Keywords:** Quantum metrology, Two-dimensional materials, Optical properties of diamond

Correction to: *Nature Communications* 10.1038/s41467-022-33399-2, published online 29 September 2022

The original version of this Article contained an error in Fig. 5, in which the labels in panels a and b were interchanged.

This has been corrected in both the PDF and HTML versions of the Article.

