# Supplementary material for: Coherent dynamics of multi-spin VB− center in hexagonal boron nitride
Source: Nat Commun. 2022 Sep 29;13:5713. doi: 10.1038/s41467-022-33399-2 (PMC9522675; doi:10.1038/s41467-022-33399-2)
Supplement: Supplementary file 1 — Supplementary Information [file 41467_2022_33399_MOESM1_ESM.pdf]

Supplementary Information: Coherent dynamics of multi-spin V_B^- centers in hexagonal boron nitride

(Dated: September 12, 2022)

Contents

Supplementary Note 1: Supplementary for experimental details 2

1. Experimental microwave setups 2
2. Pulse ODMR experimental sequences 4

Supplementary Note 2: Photoluminescence spectrum 7

Supplementary Note 3: Supplementary for ODMR spectra 8

Supplementary Note 4: Supplementary for Rabi oscillations 13

Supplementary Note 5: Supplementary for spin echo measurements 18

Supplementary Note 6: Supplementary for Ramsey measurements 20

Supplementary Note 7: Supplementary for theoretical calculation 23

Supplementary References 27

This Supplementary Information contains 27 pages and 24 figures.

Supplementary Note 1: Supplementary for experimental details

1. Experimental microwave setups

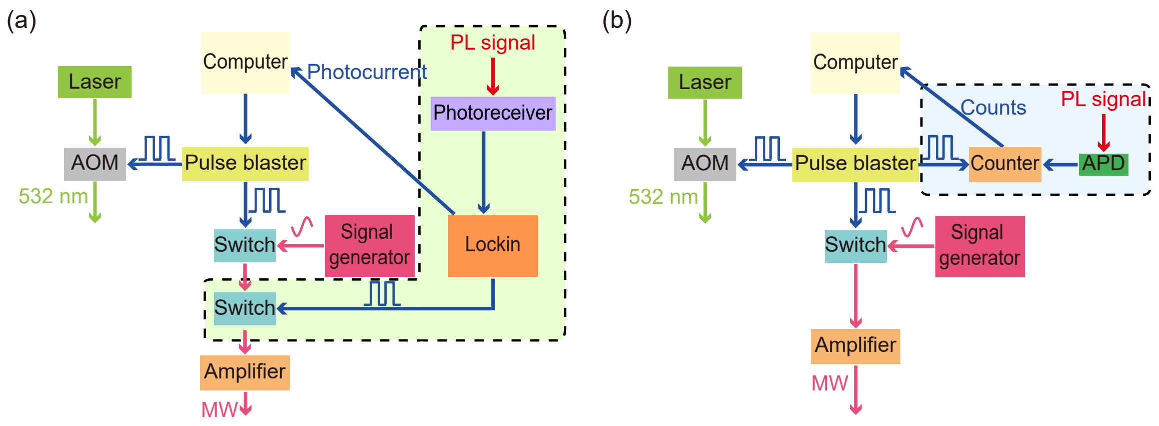

Supplementary Figure 1. Schematic diagrams of the experimental microwave (MW) systems. A 532-nm laser modulated by an acousto-optic modulator (AOM) is used for the spin initialization and excitation. The MW is generated by a synthesized signal generator which is controlled by a switch. Both the AOM and MW switch are controlled by the electrical pulse produced by a pulse blaster. In this work, we use two experimental microwave setups, called (a) Lockin setup and (b) Nicount setup. (a) The photoluminescence (PL) signal of V_B^- ensembles is detected by a photorecevier with high gain ($\sim 10^{10}$), and then converted as photocurrent and transmitted to a lock-in amplifier for the analysis and signal acquisition. The lock-in amplifier also controls an additional MW switch. In the following we call this setup as Lockin setup. (b) The PL signal of V_B^- ensembles is detected by an avalanche photodiode (APD), and then converted to electrical pulses and transmitted to a data acquisition card (USB-6341, National Instruments) for the acquisition of detected photons number. In the following we call this setup as Nicount setup.

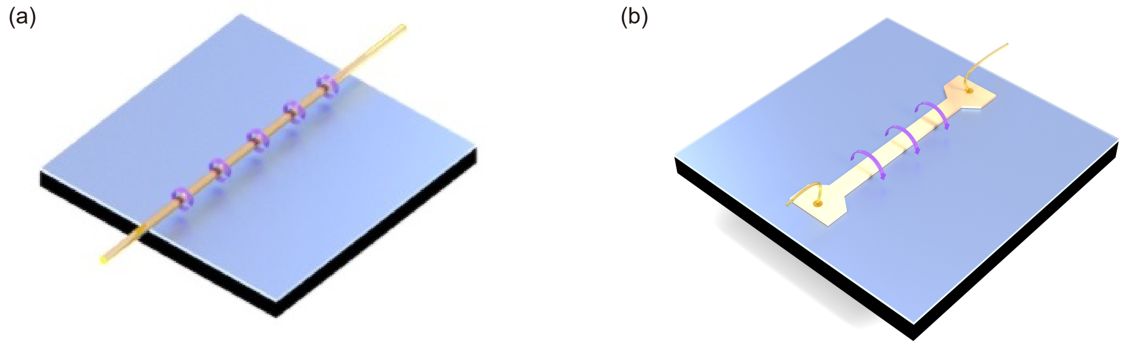

Supplementary Figure 2. Schematic representation of the experimental configuration for microwave radiation. In this work, we use two microwave radiation devices called (a) Copper-wire device and (b) Gold-stripline device. (a) A 20-μm diameter copper wire is suspended near the sample ($\sim$ μm away) and serves as an antenna to transmit the microwave field. (b) A 50-μm wide gold film microwave stripline is fabricated lithographically over the substrate by e-beam evaporation is used to transmit the microwave the field.

2. Pulse ODMR experimental sequences

All the pulse ODMR sequences in the experiments comprise two parts, 'On' and 'Off', and the schematic diagrams of pulse sequences shown in the main text are the core 'On' part. The complete pulse ODMR sequences with specific experimental details are shown in Supplementary Figs. 3-6, and the experimental signals are obtained by the On-part signals after subtracting the Off-part signals.

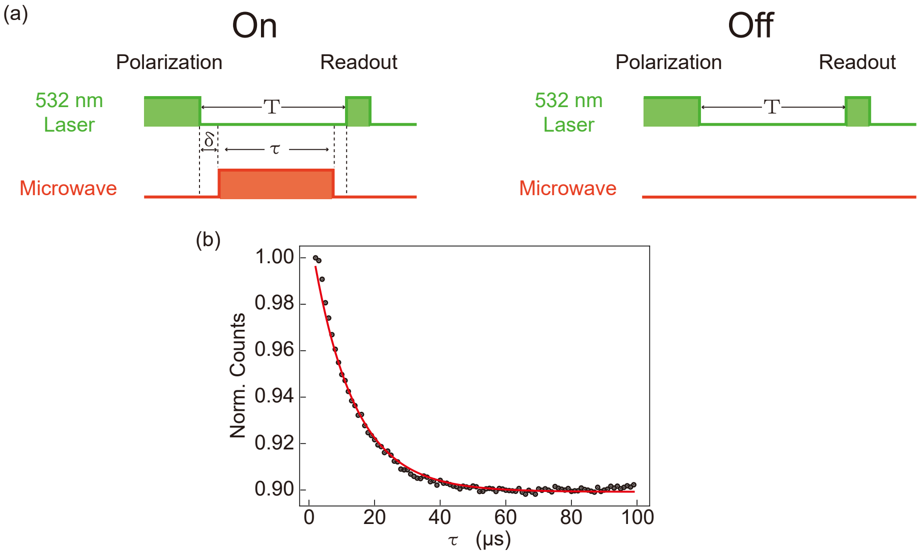

Supplementary Figure 3. Pulse sequence of Rabi oscillations. (a) The On part comprises a first laser pulse with length 3 μs for spin polarization, a microwave pulse with length τ for spin manipulation, and a second laser pulse for state readout. The time interval δ between the first laser pulse and the microwave pulse is fixed as 1 μs for the electronic spins to fully return to the ground state. The time interval between the microwave pulse and the second laser pulse varies with τ to ensure that the total time (T) is a fixed value. The Off part comprises only two laser pulses like On part, with a same delay time (T). The second laser pulse, different in the two experimental setups, is 3 μs in Lockin setup and 0.55 μs in Nicount setup. (b) A typical signal obtained by the Off pulse sequence.

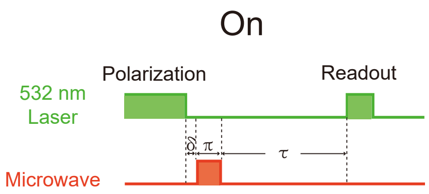

Supplementary Figure 4. Pulse sequence of T_1 measurement. The On part comprises a first laser pulse with length 3 μs for spin polarization, a microwave π-pulse for spin manipulation, and a second laser pulse for state readout. The time interval δ between the first laser pulse and the microwave pulse is fixed as 1 μs for the electronic spins to fully return to the ground state. The time interval (τ) between the microwave pulse and the second laser pulse varies from 0.01 μs to 100.01 μs. The Off part comprises only two laser pulses like On part, with the same delay. The second laser pulse is different in the two experimental setups, it is 3 μs in Lockin setup and 0.55 μs in Nicount setup.

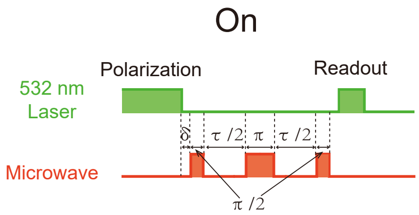

Supplementary Figure 5. Pulse sequence of spin echo measurement. The On part comprises a first laser pulse with length 3 μs for spin polarization, a microwave $\pi/2 - \pi - \pi/2$-pulses for spin manipulation, a the second laser pulse for state readout. The time interval δ between the first laser pulse and the microwave pulse is fixed as 1 μs for the electronic spins to fully return to the ground state. The time intervals between three microwave pulses are $\tau/2$, which ranges from 0.01 μs to 0.41μs. The Off part comprises only two laser pulses like On part, with the same delay. The second laser pulse is different in the two experimental setups, it is 3 μs in Lockin setup and 0.55 μs in Nicount setup.

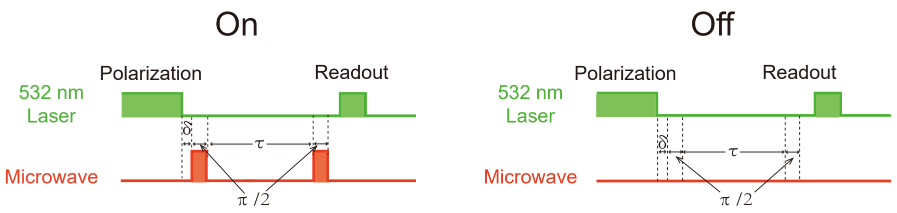

Supplementary Figure 6. Pulse sequence of Ramsey interference. The 'On' part comprises a first laser pulse with length 3 μs for spin polarization, a microwave $\pi/2-\pi/2$-pulses for spin manipulation, and a second laser pulse for state readout. The time interval δ between the first laser pulse and the microwave pulse is fixed as 1 μs for the electronic spins to fully return to the ground state. The time interval (τ) between the microwave pulses varies from 0.01 μs to 1.01 μs. The 'Off' part comprises only two laser pulses like On part, with the same delay. The second laser pulse is different in the two experimental setups, it is 3 μs in Lockin setup and 0.55 μs in Nicount setup.

Supplementary Note 2: Photoluminescence spectrum

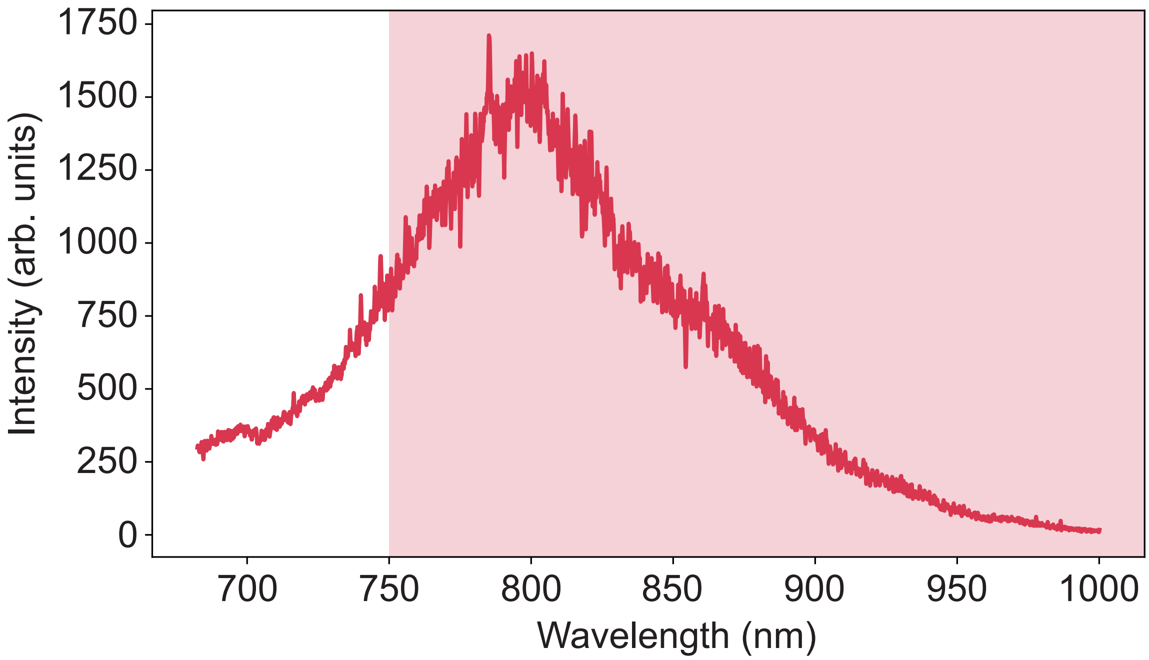

Supplementary Figure 7. Room-temperature photoluminescence (PL) spectrum of neutron-irradiated hBN sample. The PL has been filtered by a 600-nm long-pass filter (FELH0600, Thorlabs), and the PL in the coherent manipulation experiment is filtered by a 750-nm long-pass filter (FELH0750, Thorlabs).

Supplementary Note 3: Supplementary for ODMR spectra

We generate the magnetic fields along c axis with different intensities by electromagnets, and measure the ODMR spectra of V_B^- defect in hBN under different magnetic fields by the Lockin setup (Supplementary Fig. 8). The results in Fig. 1(d) in the main text are obtained from these data shown in Supplementary Fig. 8. We fit the ODMR spectra under different magnetic fields by a seven Lorentz function and obtain the hyperfine structures shown in Supplementary Fig. 9. The fitting results show that the splitting constant $A \sim 45$ MHz, which is consistent with the previously reported V_B^- in other works [1]. In addition, we also observe an obvious enhancement of the nuclear spin polarization with the increase of magnetic-field intensity as shown in Supplementary Fig. 10.

According to the fitted hyperfine structures shown in Supplementary Fig. 9, we find that the broadening of hyperfine peaks is not changed with the magnetic field. To further verify the broadening of hyperfine peaks under strong magnetic field, we measure the ODMR spectra under strong magnetic fields in Lockin setup and Nicount setup, with different MW powers and excitation-laser powers. Supplementary Figure 10 shows the ODMR spectra of V_B^- defects in hBN sample measured at 3-mW laser and 39-mT magnetic field. When the microwave power decreases, no trend of narrowing of the hyperfine peaks is seen. Supplementary Figure 11 shows the ODMR spectra of V_B^- defects in hBN measured at 100-μW laser and 45-mT magnetic field. When the microwave power decreases to 0.05 mW, no narrowing trend of the hyperfine peaks is seen until the signal is submerged in the nosie. It indicates that the hyperfine peaks in the ODMR cannot sharpen even with low microwave power and low excitation power in this work. In principle, the broadening of hyperfine peaks is due to the hyperfine interaction with the boron atoms in the second neighbor shell of V_B^- center.

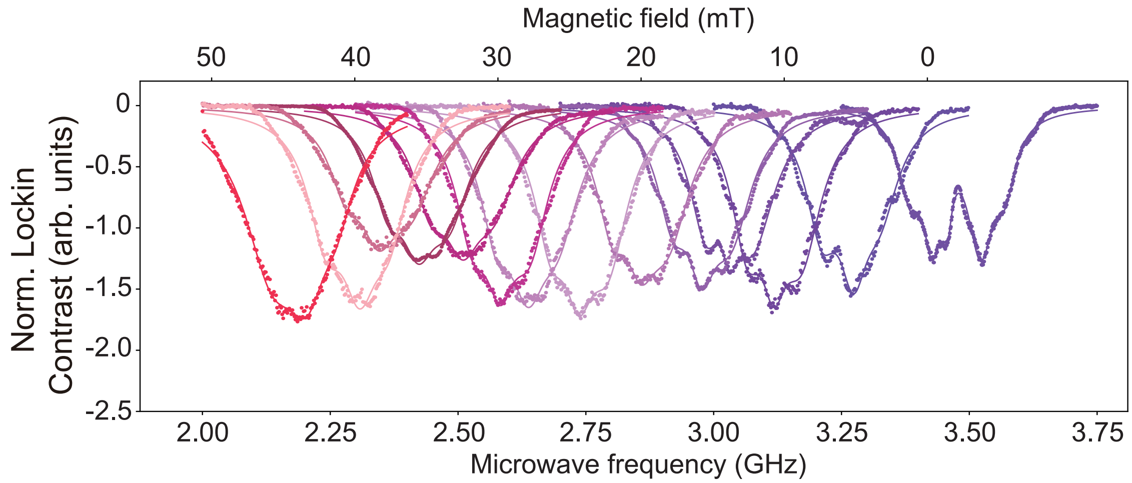

Supplementary Figure 8. Magnetic-field dependence of the ODMR spectrum. ODMR spectra of the V_B^- defect in hBN are measured under various magnetic fields and fixed 3-mW laser. The depicted ODMR spectra (except 0-mT ODMR spectrum) correspond to only the transition between $m_s = -1$ and $m_s = 0$ spin states.

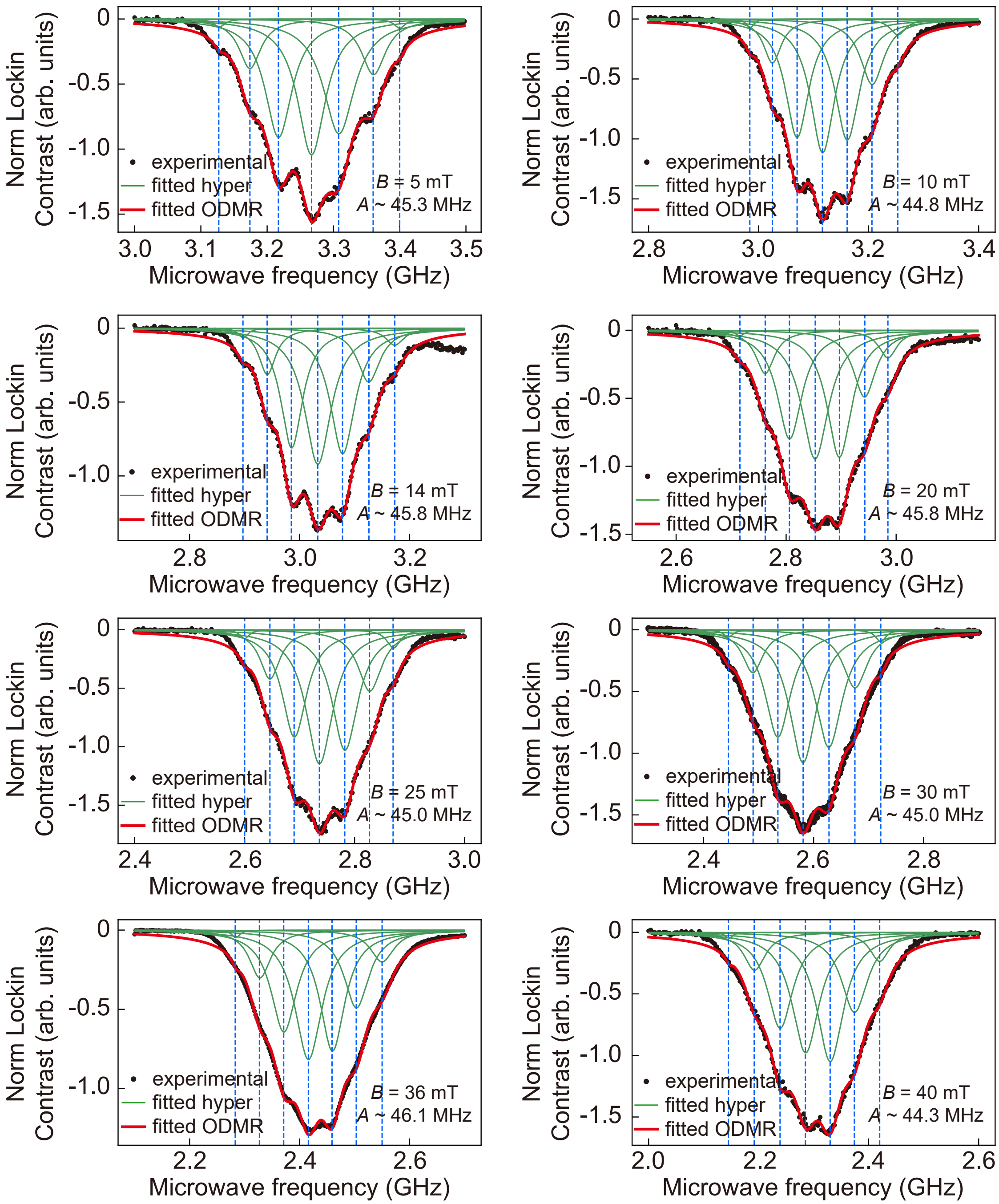

Supplementary Figure 9. Analysis of the hyperfine structure. ODMR spectra are recorded at different magnetic fields, and fitted with a seven Lorentz function (red solid curves), to obtain the seven hyperfine components (green solid curves) and hyperfine peaks (blue dashed lines). The obtained characteristic hyperfine splitting is $A \sim 45.3$ MHz.

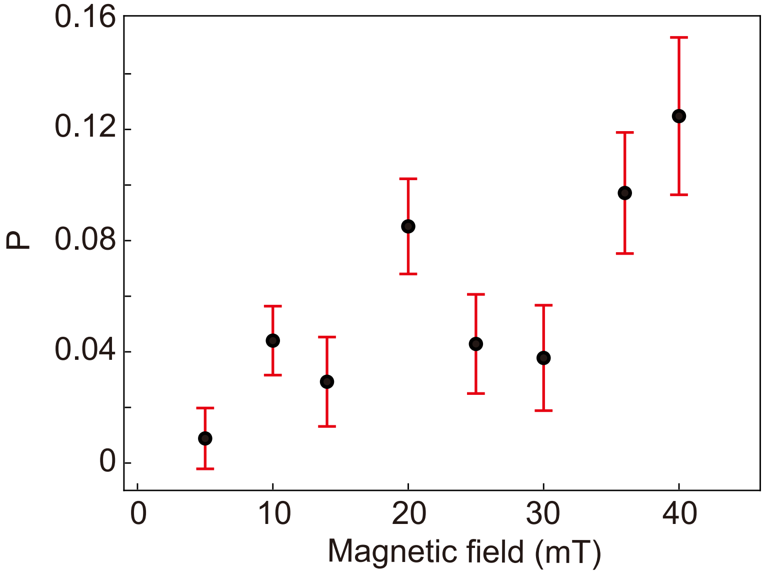

Supplementary Figure 10. The average polarization P of the three nearest neighbor ^{14}N nuclear spins at different magnetic fields where $P = \Sigma_{m_I} m_I \rho_{m_I} / (3\Sigma_{m_I} \rho_{m_I})$. The summation is performed over the seven hyperfine peaks in the ODMR spectra, ρ_{m_I} denotes the fitted relative population on corresponding m_I nuclear spin state.

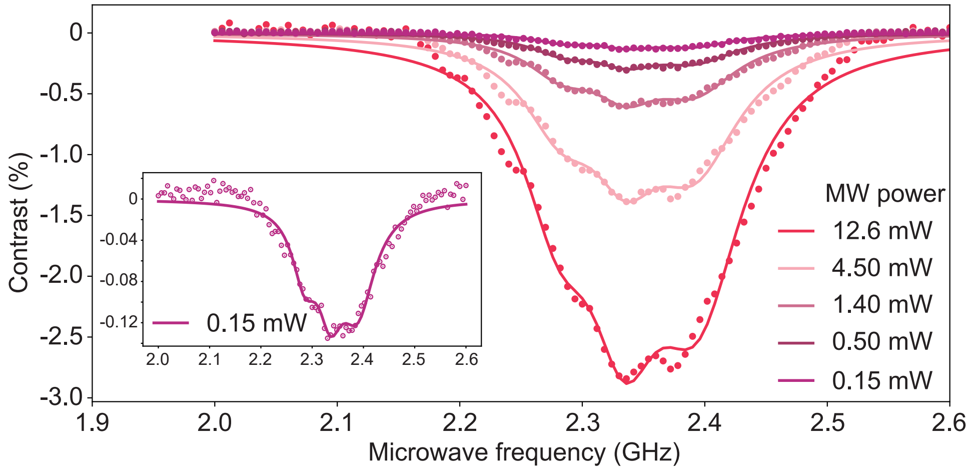

Supplementary Figure 11. Microwave-power dependence of the ODMR spectrum. ODMR spectra of the V_B^- defects in hBN sample are measured under different microwave powers and fixed 3-mW laser power at 39-mT magnetic field.

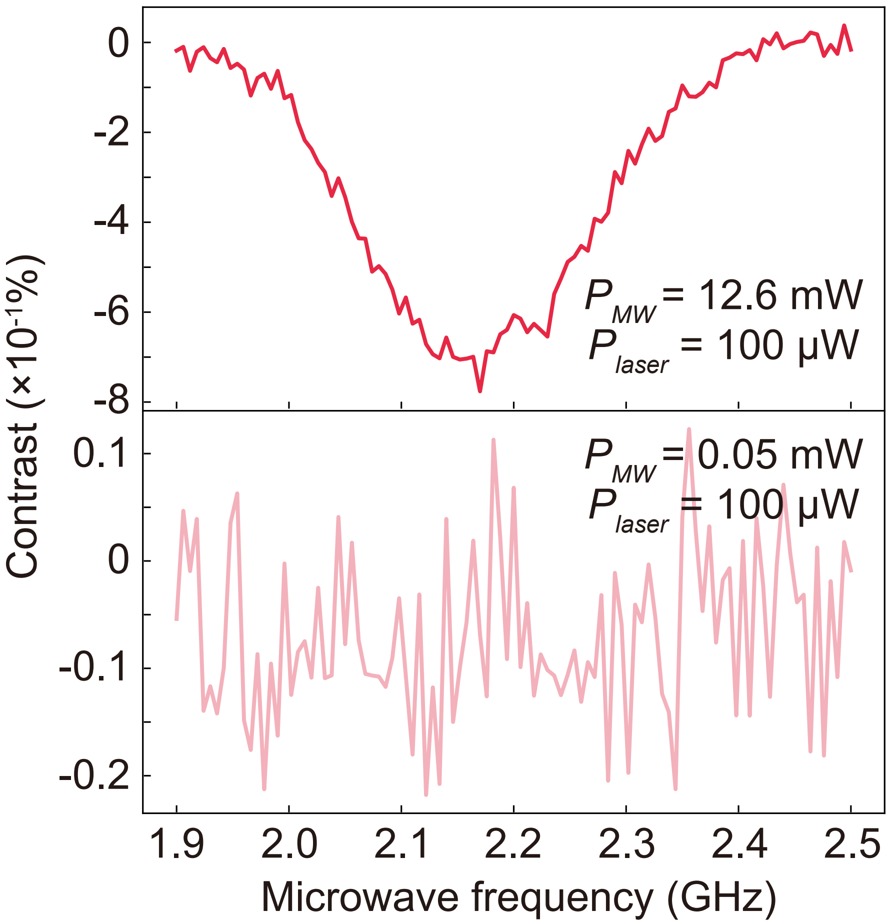

Supplementary Figure 12. ODMR spectra of the V_B^- defect in hBN sample measured at 100-μW laser power and 45-mT magnetic field. The ODMR signal is already very weak and submerged in noise when the MW power drops to 0.05 mW.

Supplementary Note 4: Supplementary for Rabi oscillations

For the Rabi oscillations shown in Fig. 2(b) in the main text, we use the following fitting functions:
B =0 mT (n = 1) :

$$f(\tau) = ae^{-\tau/T_a}\cos(2\pi f_1\tau + \phi_1) + be^{-\tau/T_b} + c, \tag{1}$$

B =16 mT (n=2):

$$f(\tau) = ae^{-\tau/T_a}\prod_{i=1}^{n=2}\cos(2\pi f_i\tau + \phi_i) + be^{-\tau/T_b} + c, \tag{2}$$

B =44 mT (n=3):

$$f(\tau) = ae^{-\tau/T_a}\prod_{i=1}^{n=3}\cos(2\pi f_i\tau + \phi_i) + be^{-\tau/T_b} + c, \tag{3}$$

and the fitting results are
B =0 mT : $f_1 = 19.54$ MHz

$$f(\tau) = 0.005e^{-\tau/0.038}\cos(122\tau - 0.014) + 0.0013e^{-\tau/0.166} - 0.009, \tag{4}$$

B =16 mT : $f_1 = 14.90$ MHz; $f_2 = 3.57$ MHz

$$\begin{aligned} f(\tau) = &-0.003e^{-\tau/0.04}\cos(93.6\tau - 0.575)\cos(22.4\tau + 3.415) \\ &+ 0.0005e^{-\tau/0.52} - 0.006, \end{aligned} \tag{5}$$

B =44 mT : $f_1 = 11.21$ MHz; $f_2 = 1.96$ MHz; $f_3 = 0.012$ MHz

$$\begin{aligned} f(\tau) = &\,0.082e^{-\tau/0.105}\cos(70.4\tau + 0.124)\cos(12.3\tau + 0.381)\cos(0.075\tau + 1.555) \\ &+ 0.0004e^{-\tau/0.069} - 0.003. \end{aligned} \tag{6}$$

Here n is the amount of the different oscillation components utilized in the fitting function. It can be seen that the oscillation decay time T_a in the fitting results increases with the

magnetic field intensity, which reveals that the V_B^- spin relaxation can be suppressed at strong magnetic field.

We measure the Rabi oscillations of V_B^- spins at 11.5-mT magnetic field with different MW powers (Supplementary Fig. 13) and MW detunings (Supplementary Fig. 14). The red solid curves in Supplementary Fig. 13 and 14 are the fitting results with the single-frequency fitting function $f(\tau) = ae^{-\tau/T_a}\cos(2\pi f\tau + \phi) + be^{-\tau/T_b} + c$, where f is the Rabi frequency. The Rabi-frequency dependence on MW powers shown in Fig. 2(c) in the main text corresponds to the fitting results of Supplementary Fig. 13. We measure the Rabi oscillations with varying MW detunings at fixed magnetic field (11.5 mT) and MW power (5.8 W) (Supplementary Fig. 14(b)). There is no obvious change for the Rabi-oscillation frequency under different MW detunings, but only the reduction of Rabi-oscilition amplitude with the MW detunings. Likewise, we perform a similar measurement at 22 mT and obtain similar results (Supplementary Fig. 14(d)), while the multiple-frequency oscillation character emerges at 22 mT. In addition, we measure Rabi oscillations at different positions on the hBN sample, and they exhibit similar Rabi oscillation characters, indicating that the Rabi oscillations we measured are characteristic for the entire sample (Supplementary Fig. 15). We need to point out that the slight difference in Rabi oscillations here is mainly caused by the local variation of MW power related to the positions on the gold film MW stripline.

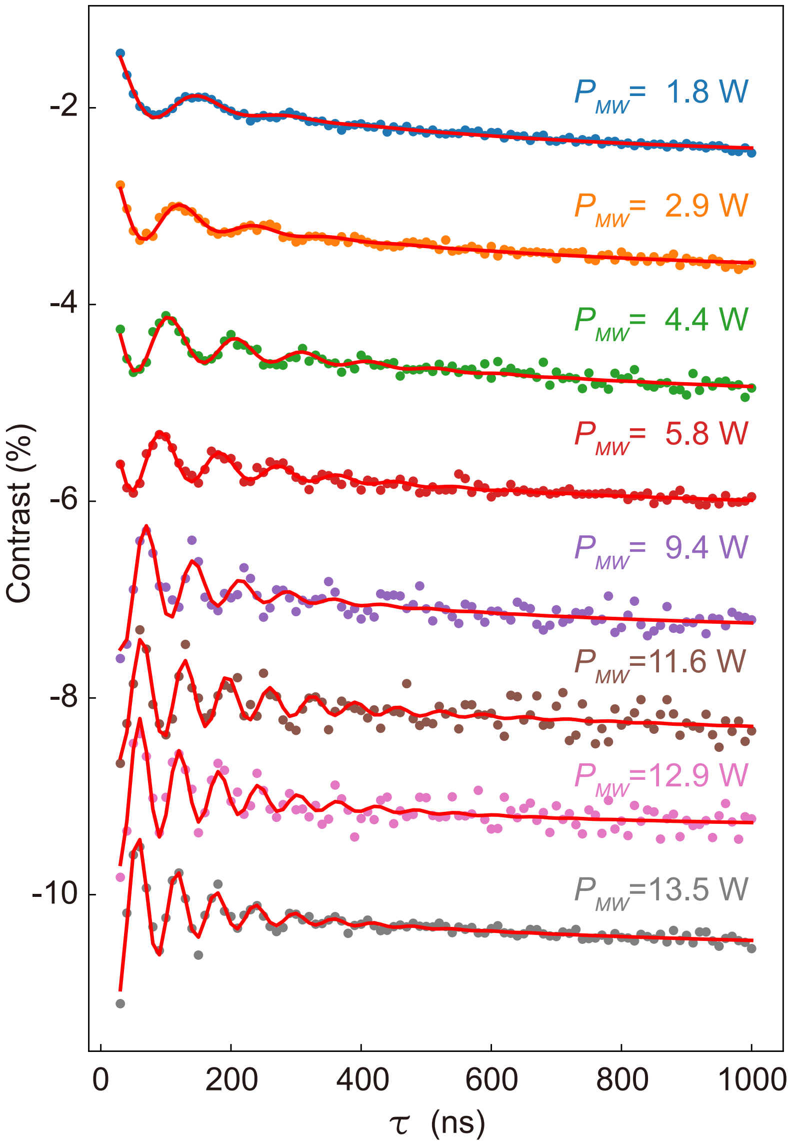

Supplementary Figure 13. Microwave-power-dependent Rabi oscillations measured at 11.5 mT magnetic field and 3100 MHz microwave frequency. The red solid curves are the fitting results with the fitting function $f(\tau) = ae^{-\tau/T_a}\cos(2\pi f\tau + \phi) + be^{-\tau/T_b} + c$.

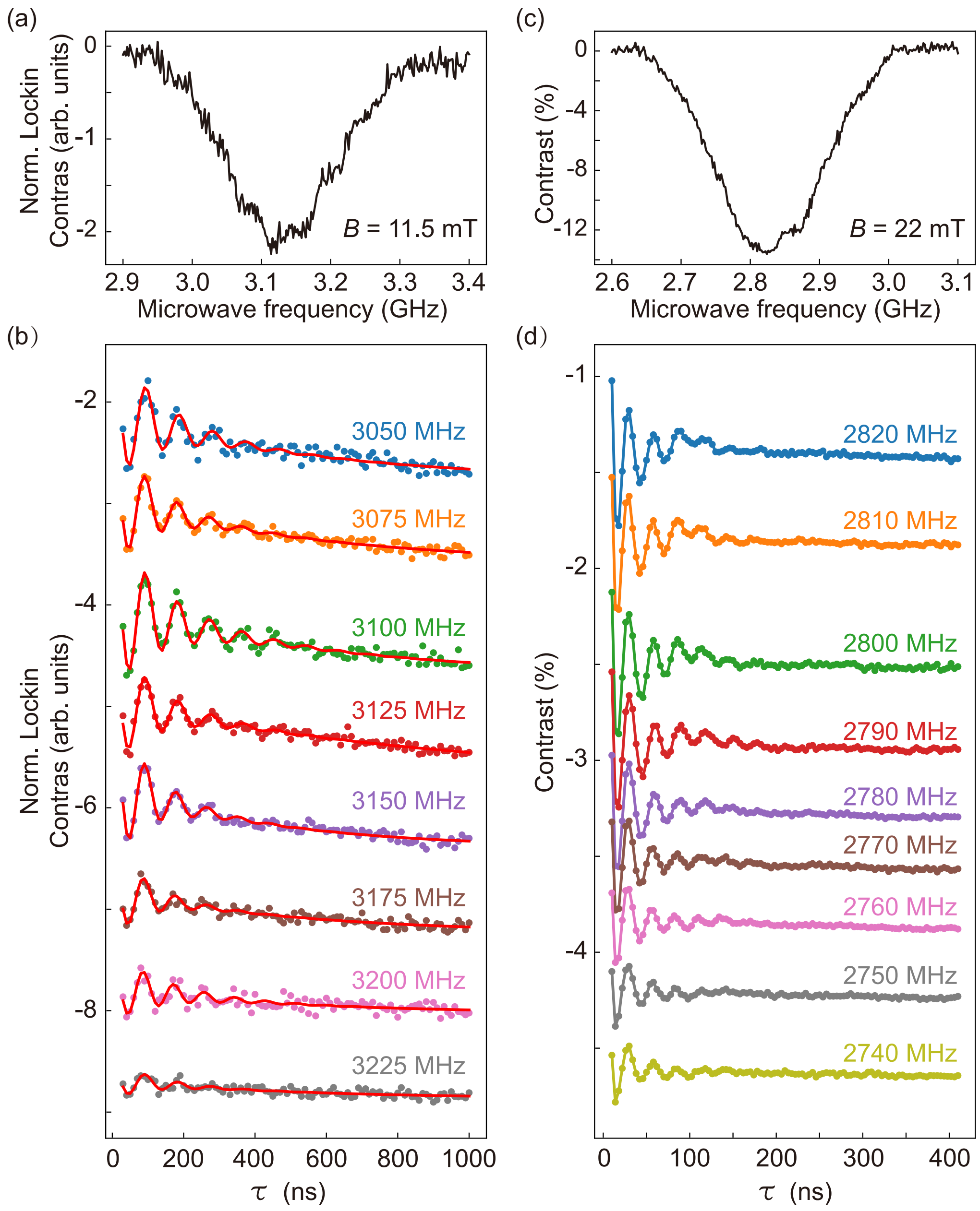

Supplementary Figure 14. Microwave-detuning-dependent Rabi oscillations. (a) ODMR spectrum measured at 11.5 mT magnetic field. (b) Microwave-detuning-dependent Rabi oscillations measured at 11.5 mT magnetic field and 5.8 W microwave power. The red solid curves are the fitting results with fitting function $f(\tau) = ae^{-\tau/T_a}\cos(2\pi f\tau + \phi) + be^{-\tau/T_b} + c$. (c) ODMR spectrum measured at 22 mT magnetic field. (d) Microwave-detuning-dependent Rabi oscillations measured at 22 mT magnetic field.

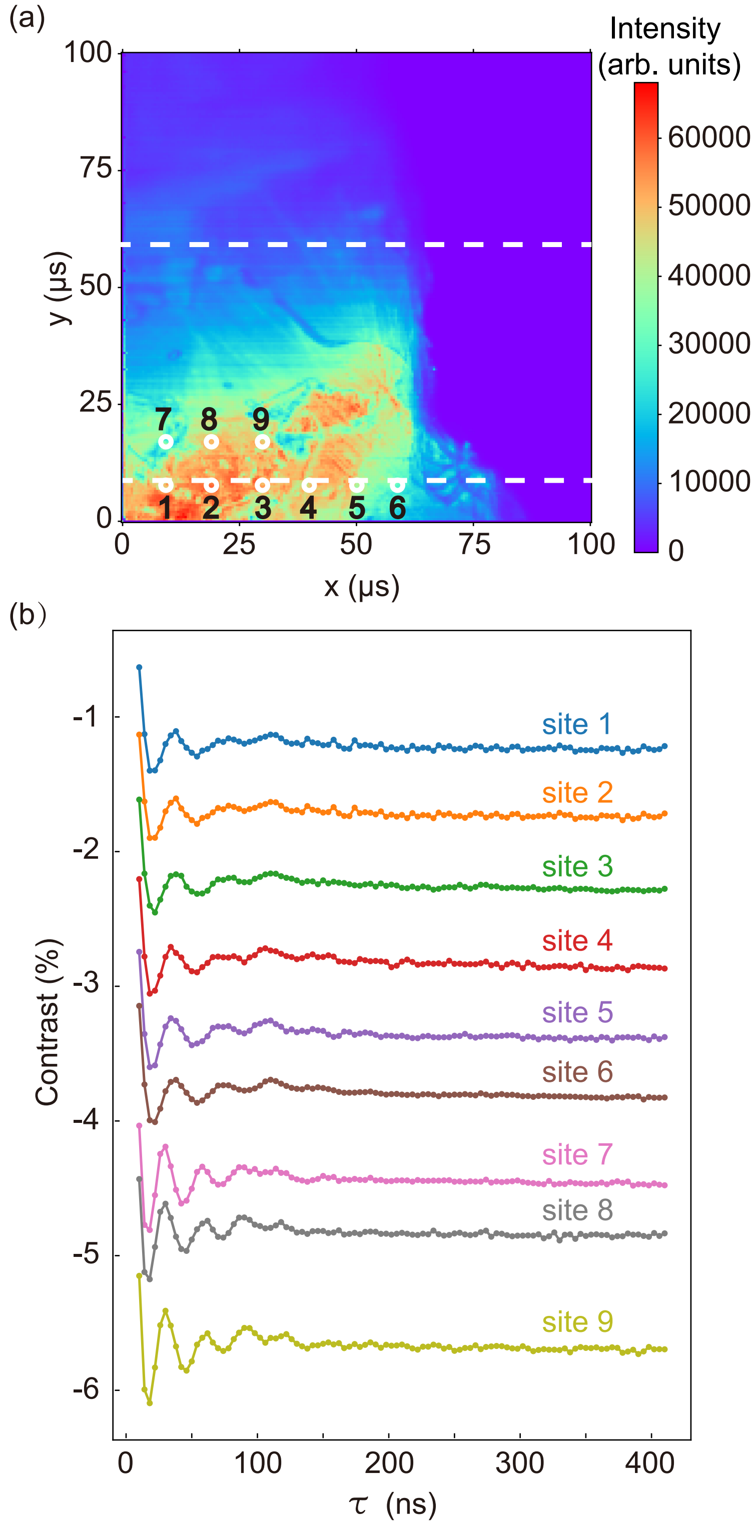

Supplementary Figure 15. Rabi oscillations at different sites on hBN sample. (a) Photoluminescence (PL) map for the neutron-irradiated hBN bulk sample. The nine white circles represent the nine positions where Rabi oscillations are tested in the experiment. The area in the middle of the white dashed lines is the gold electrode (the position is not exact, just for reference). (b) Rabi oscillations at different positions on the hBN sample at 22 mT.

Supplementary Note 5: Supplementary for spin echo measurements

At 36-mT magnetic field, the decayed-contrast curve of spin echo becomes a complicatedly modulated curve as shown in Fig. 3(f) in the main text, which cannot be fitted well. We repeat the 36-mT spin echo measurement for many times, and the obtained results show similar tendency beyond the error bars as shown in Supplementary Fig. 16. It indicates that the complicatedly modulated curves are not noise. Supplementary Figure 17 shows the spin echo at other magnetic fields of 24 and 30 mT. The 24-mT and 30-mT spin echoes are not standard exponential decaying curves, and also exhibit slightly complicated oscillations. As the magnetic field increases, these oscillations seem to have a tendency to become more complicated. The complicated modulation of the spin echo should be related to the strong electronic-nuclear spin coupling existing in V_B^- center and the suppression of spin relaxation at strong magnetic field.

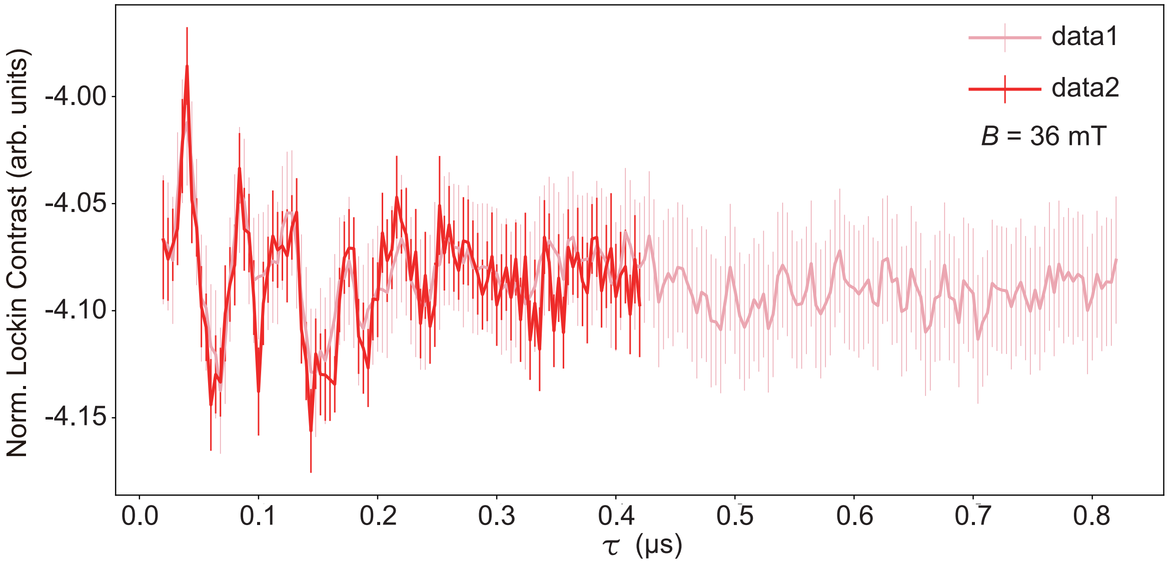

Supplementary Figure 16. Spin echo at 36-mT magnetic field with error bars. The light-red curve is the data corresponding to Fig. 3(f) in the main text.

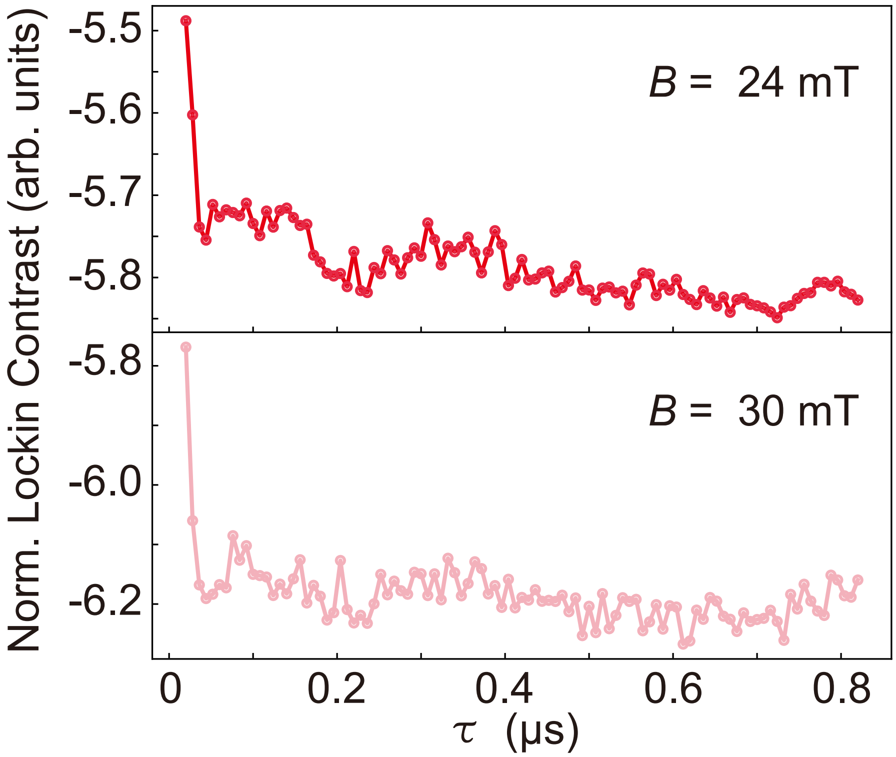

Supplementary Figure 17. Spin echo at magnetic fields of 24 and 30 mT. The observed spin echo exhibit complicated modulation curves at strong magnetic fields.

Supplementary Note 6: Supplementary for Ramsey measurements

Supplementary Figure 18 shows the Ramsey result at 0-mT magnetic field and 3428-MHz driving MW. No oscillation is observed but a fast decay with a slow background decay. We use the fitting function $ae^{-\tau/T_a} + be^{-\tau/T_b} + c$ to fit the Ramsey curves in Supplementary Fig. 18, and the fitting result is $0.001e^{-\tau/0.060} + 0.000007e^{-\tau/0.376} - 0.012$. Noted that the exact 0-mT T_2^* here should be very short, and the V_B^- spin would be already decoherent during the first $\pi/2$-pulse, hence this 0-mT Ramsey result could not accord with the standard Ramsey measurement. In addition, the Ramsey results at different magnetic fields are shown in Supplementary Fig. 19, and the Ramsey results with different MW detunings are shown in Supplementary Fig. 20. It is difficult to give a strict relationship of the Ramsey oscillation with the magnetic field, but it is certain that as the magnetic field becomes strong, the Ramsey oscillation shows to contain a significant multiple-frequency component, which should be related the MW-driven V_B^- spins at the hyperfine states.

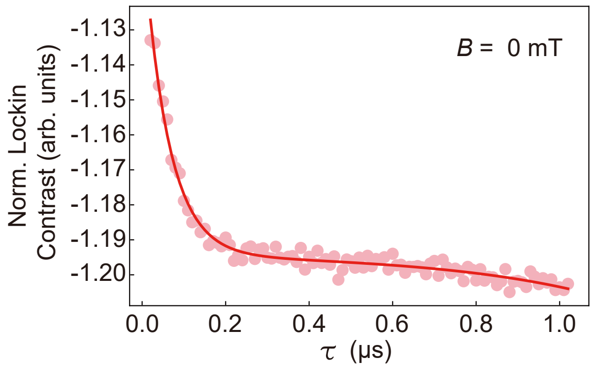

Supplementary Figure 18. Ramsey result at 0-mT magnetic field and 3428-MHz driving MW. The red curve is the fitting result of the experimental results (red dots).

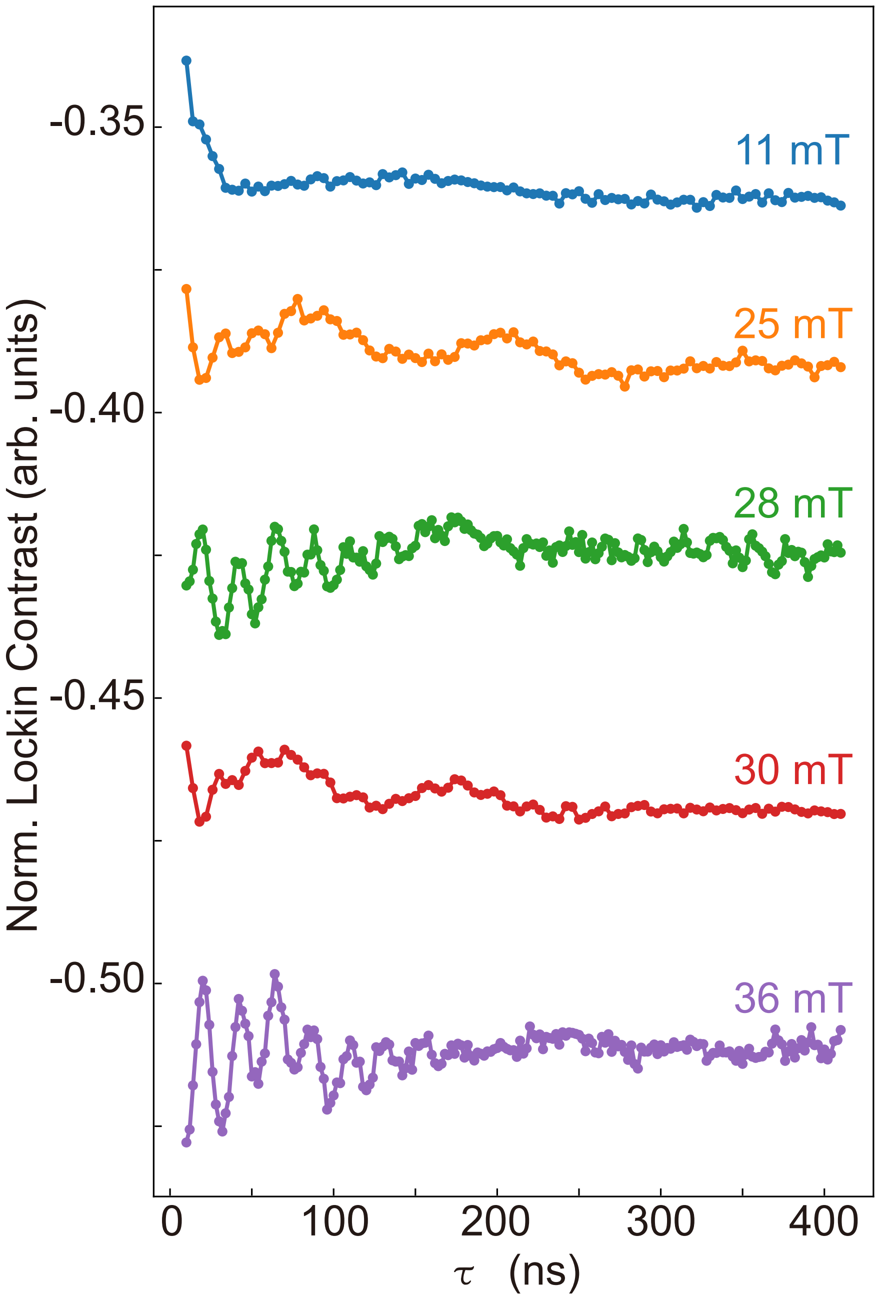

Supplementary Figure 19. Ramsey results at different magnetic fields. The blue, orange, green, red and purple curves show the observed Ramsey results at magnetic fields of 11 mT, 25 mT, 28 mT, 30 mT and 36 mT, respectively.

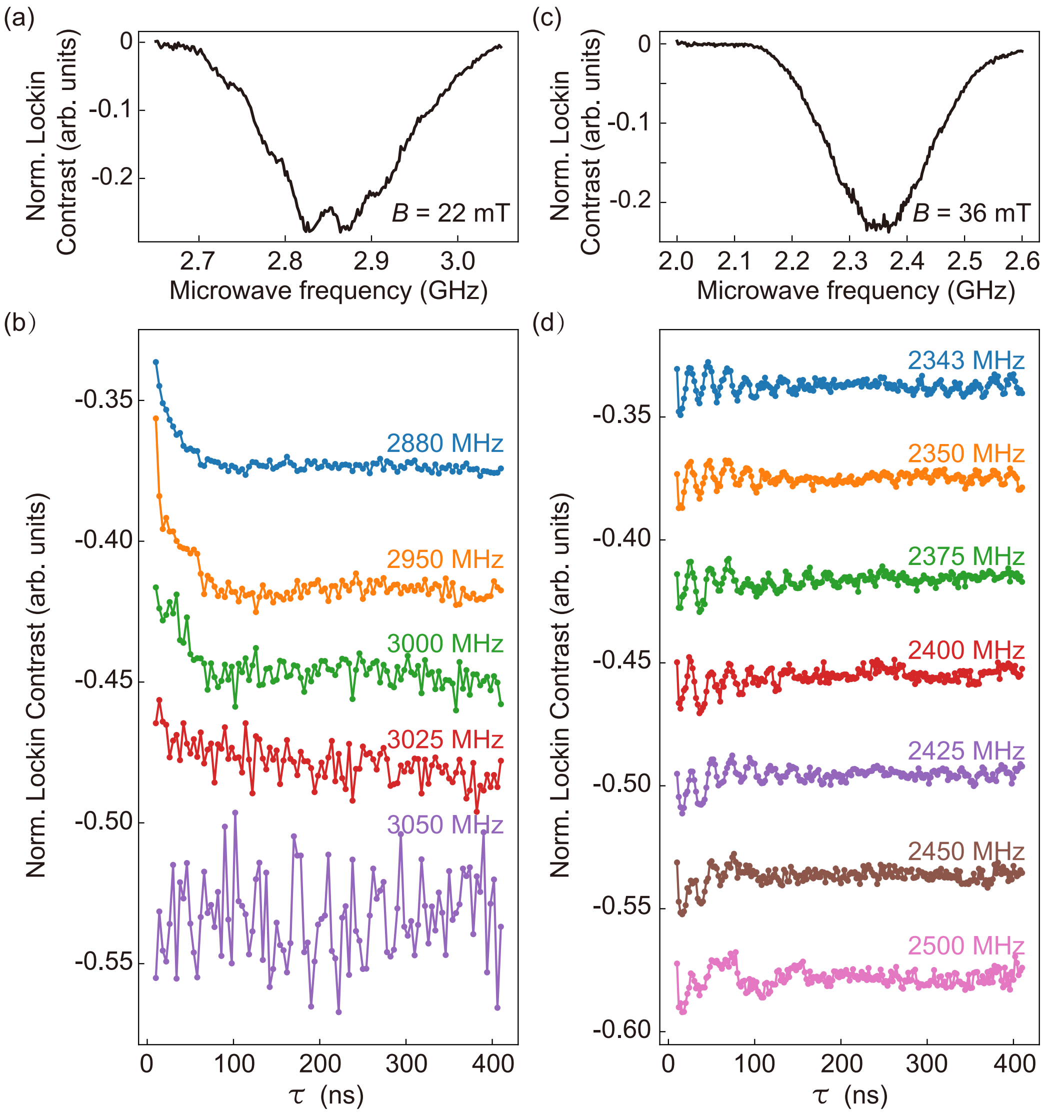

Supplementary Figure 20. Ramsey results driven by the MW with different detunings. (a) ODMR spectrum measured at 20-mT magnetic field. (b) Ramsey results with different MW detunings at 20 mT. (c) ODMR spectrum measured at 36-mT magnetic field. (d) Ramsey results with different MW detunings at 36 mT.

Supplementary Note 7: Supplementary for theoretical calculation

In order to study the spin dynamics of a MW driven V_B^- system, we use (1) exact time evolution of a closed 4-spin model consisting of one 1-spin electron and its three nearest neighbor 1-spin ^{14}N, and (2) a cluster approximation based extended Lindbladian method [2]. In contrast to method (1), method (2) can induce relaxation effects in a parameter-free manner. In both methods, the MW field is added with no approximation, i.e. an oscillating magnetic field with in-plane magnetic field polarization is added in the model to describe the external drive. This allows us to account for dressing and multi-spin resonances. In the simulations of the 4-spin V_B^- system, the system Hamiltonian is

$$H = D_{\mathrm{gs}}[S_z^2 - S(S+1)/3] + \sum_{k=1,2,3} \mathbf{S}\mathbf{A_k}\mathbf{I_k} + \gamma_e B_0 S_z - \sum_{k=1,2,3} \gamma_n B_0 I_{zk} + \sum_{k=1,2,3} Q_j (I_{zk}^2 - I_k(I_k+1)/3)$$

where D_{gs} is zero-field splitting (ZFS), $\mathbf{S}$ and $\mathbf{I}$ are the spin-1 electronic and nuclear operators, respectively, $\mathbf{A}$ is the hyperfine-interaction tensor, γ_e (γ_n) is the electronic (nuclear) spin gyromagnetic ratio, Q_j is the bulk quadrupole parameter.

The *ab initio* Hamiltonian parameters used in our calculation are: $D_{\mathrm{gs}} = 3471\mathrm{MHz}$, ^{14}N quadrupole $Q_{^{14}\mathrm{N}} = 3 \times 0.022/4 = 0.165$, ^{11}B quadrupole $Q_{^{11}\mathrm{B}} = 3 \times 3.717/12 = 0.929$. The magnetic field is set to 21.7 mT. The hyperfine parameters of the nearest neighbor ^{14}N nuclear spins are:

$$A_{1,xx,gs} = 80.219; A_{2,xx,gs} = 46.110; A_{3,xx,gs} = 80.202$$

$$A_{1,yy,gs} = 57.486; A_{2,yy,gs} = 91.571; A_{3,yy,gs} = 57.479$$

$$A_{1,zz,gs} = 47.957; A_{2,zz,gs} = 47.935; A_{3,zz,gs} = 47.935$$

$$A_{1,xy,gs} = 19.687; A_{2,xy,gs} = -0.004; A_{3,xy,gs} = -19.687$$

$$A_{1,xz,gs} = 0.0; A_{2,xz,gs} = 0.0; A_{3,xz,gs} = 0.0$$

$$A_{1,yz,gs} = 0.0; A_{2,yz,gs} = 0.0; A_{3,yz,gs} = 0.0$$

In the simulation of ODMR spectrum, we focus on the closed 4-spin V_B^- model, and the results are shown in Fig. 1(f) in the main text. The driving MW is simulated with the sweeping resolution of 20 MHz. Since the closed system includes only first neighbors

and the MW broadening is smaller than the hyperfine splitting, the hyperfine structure of the ODMR signal can be resolved in the simulations. The central peak in our simulations, with the above-mentioned parameters, is found at 2878 MHz. Note that the inclusion of spin polarization of the nearest neighbor ^{14}N nuclear spins changes the relative intensities of hyperfine peaks in ODMR spectrum. Therefore, in a polarized environment the largest amplitude peak may not correspond to the central peak, as the ODMR spectra shown in Fig. 1(f) in the main text. In addition, the driving MW itself also gives rise to a polarization of the surrounding spin bath. As can be seen in Supplementary Fig. 21, the induced polarization depends on the detuning of the driving frequency from the central peak of 2878 MHz. For the low-frequency detuning, the ^{14}N nuclear spins are polarized in the m_I =+1 state, while for the high-frequency detuning, the polarization tends to in the m_I =-1 state. At the center with no detuning, the polarization pattern is mixed.

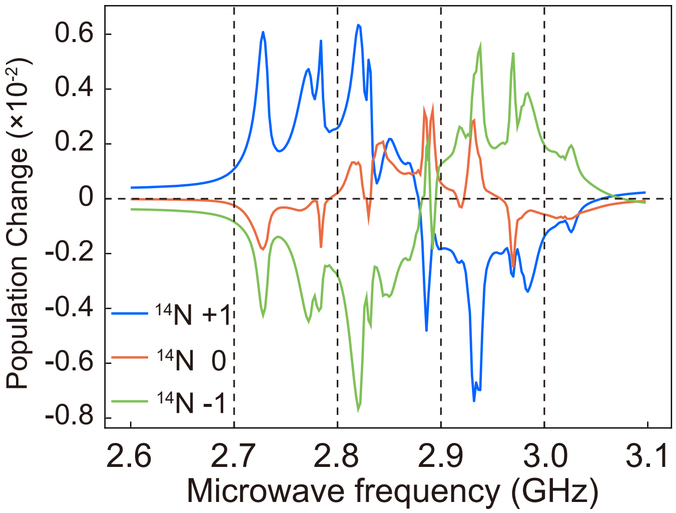

Supplementary Figure 21. Nearest neighbor ^{14}N nuclear spin polarization induced by the MW drive. The red, green, blue curves show the change of the polarization of the $m_I(^{14}\mathrm{N})$ = +1, 0, and -1 spin states under continuous MW drive.

In the simulation of Rabi oscillation, we first study the closed 4-spin system in detail, and found that the Rabi oscillation does not decay in this model as shown in Fig. 2(e) in the main text. Due to the involvement of the ^{14}N nuclear spins in the oscillations, the alternating curve is not a simple cosine function. As the driving frequency is detuned from the central peak frequency, the oscillation amplitude reduces and the oscillation frequency

increases, and the waveform gets further distorted. In the Rabi oscillation, the ^{14}N nuclear spins in V_B^- are also driven by the MW. As can be seen in Fig. 2(d) in the main text, the first neighbor ^{14}N nuclear spins oscillate with nearly the same frequency as the electron spin. At the same time, they acquire a net polarization with time. In Supplementary Fig. 22, we depict the Rabi oscillations that are obtained with an initially unpolarized or polarized first neighbor shell. It can be seen that the initial polarization of ^{14}N nuclear spins have only a minor effect on the Rabi-oscillation curves.

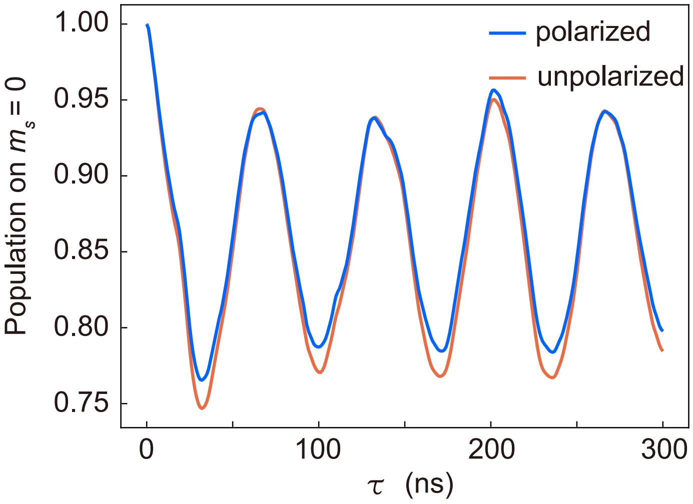

Supplementary Figure 22. Rabi oscillations with the initially unpolarized or polarized nearest neighbor ^{14}N nuclear spins. The blue and red curves show the simulated V_B^- Rabi oscillations with an polarized and unpolarized first neighbor ^{14}N nuclear spin shells, respectively. The Rabi oscillations change little with the polarization of first neighbor nuclear spin shell.

Next, we perform many-body simulations with cluster approximation. We utilize our parameter free many-body approach [2] to model the Rabi oscillation of the open V_B^- model. We consider the effects of a many-body nuclear spin bath, containing 127 ^{14}N or 127 ^{11}B, on the central 4-spin V_B^- system. The results of the Rabi oscillations with relaxation effect are shown in Figs. 5 (a)&(b) in the main text. In addition, we also obtain the nearest neighbor ^{11}B and ^{14}N nuclear spin polarization dependence in the Rabi oscillation, where the interaction with the many-body ^{11}B nuclear spin bath is taken into account. The results are shown in Supplementary Figs. 23 and 24. In both cases, the polarization transfers to the nearest neighbor nuclear spin bath. However, it is much more prominent for the

nearest neighbor ^{14}N nuclear spins. Hence we conclude that the nearest neighbor ^{14}N nuclear spins are responsible for the modulation of the Rabi oscillation, including the decay of the background beyond 0.2 μs. Rest of the spin bath is responsible for the decay of the Rabi oscillation.

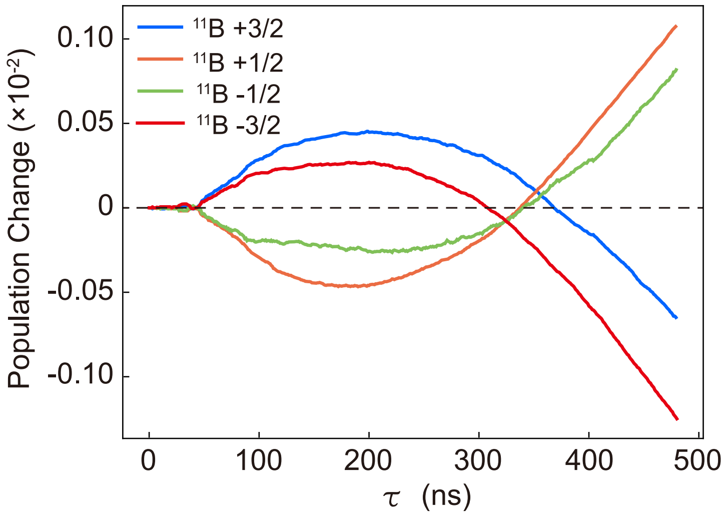

Supplementary Figure 23. Nearest neighbor ^{11}B polarization dependence in the Rabi oscillation, under interaction with the many-body ^{11}B nuclear spin bath. The blue, orange, green and red curves show the simulated population changes of ^{11}B nuclear spins on $m_I = +3/2$, $+1/2$ and -1/2 and -3/2, respectively. The total population of nearest neighbor ^{11}B nuclear spin changes little under the Rabi oscillation, compared with the nearest neighbor ^{14}N shown in Supplementary Fig. 24.

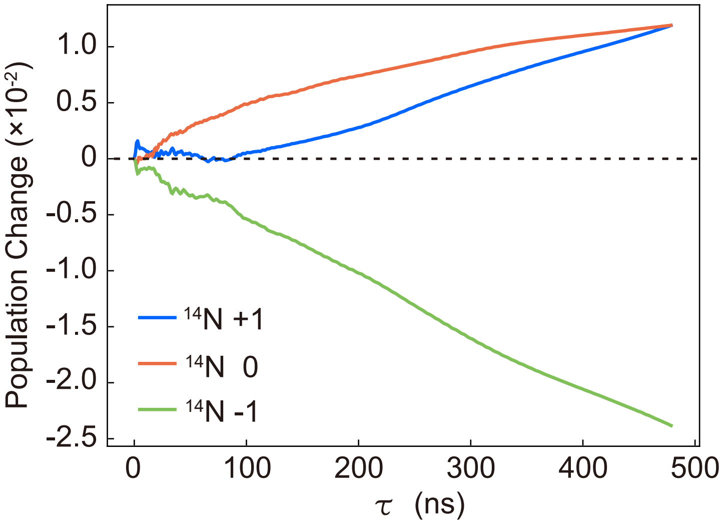

Supplementary Figure 24. Nearest neighbor ^{14}N polarization dependence in the Rabi oscillation, under interaction with the many-body ^{11}B nuclear spin bath. The blue, red and green curves show the simulated population changes of ^{14}N nuclear spins on $m_I = +1$, 0 and -1, respectively. The total population of nearest neighbor ^{14}N nuclear spin would incline to $m_I = -1$ under the Rabi oscillation.

Supplementary References

[1] Gottscholl, A. *et al.* Initialization and read-out of intrinsic spin defects in a van der Waals crystal at room temperature. *Nature Mater.* **19**, 540-545 (2020).

[2] Ivády, V. Longitudinal spin relaxation model applied to point-defect qubit systems. *Phys. Rev. B* **101**, 155203 (2020).
